# Definitions used for a healthy periodontium—A systematic review

**DOI:** 10.1111/idh.12438

**Published:** 2020-05-24

**Authors:** An Li, Renske Z. Thomas, Luc van der Sluis, Geerten‐Has Tjakkes, Dagmar Else Slot

**Affiliations:** ^1^ Center for Dentistry and Oral Hygiene University Medical Center Groningen (UMCG) University of Groningen Groningen The Netherlands; ^2^ Department of Dentistry Radboud Institute for Health Sciences Radboud University Medical Center Nijmegen The Netherlands; ^3^ Department of Periodontology Academic Centre for Dentistry Amsterdam (ACTA) University of Amsterdam and Vrije Universiteit Amsterdam Amsterdam The Netherlands

**Keywords:** definition, periodontal health, periodontium, systematic review

## Abstract

**Objective:**

To investigate the explicitness and variability of the definition of periodontal health in the current scientific literature.

**Material and methods:**

The authors conducted a systematic literature review using PubMed and CENTRAL (2013‐01/2019‐05) according to the Preferred Reporting Items for Systematic Reviews and Meta‐Analyses (PRISMA) guidelines and the guidelines of the Meta‐analysis Of Observational Studies in Epidemiology (MOOSE) statement.

**Results:**

A total of 51 papers met the predefined inclusion criteria. Of these, 13 papers did not report any explicit definitions of periodontal health. Out of the 38 remaining articles, half of them used a reference to support their definition and half of them not. The studies published in periodontics‐related journals or those that scored a low risk of bias for the methodical quality presented more explicit and valid definitions. Probing pocket depth was the most frequently used individual parameter for defining periodontal health. However, there were substantial variations in the methods of measurement and cut‐off values.

**Conclusions:**

Given the diversity of periodontal health definitions, a cross‐study comparison is difficult. The results of this review may be useful in making others aware of the significance of standardizing the definition of a healthy periodontium.

## 1 INTRODUCTION

The main objective of periodontal care is to reach and maintain a healthy periodontium. The definition of periodontal health plays a crucial role in population surveillance and the determination of critical therapeutic targets for clinicians.^1^ Most studies traditionally regarded that a healthy periodontium is the opposite of case definitions of periodontal disease, as does the World Health Organization (WHO) defining health as an absence of illness.^2^ Specifically, periodontal health refers to a state free from inflammation and characterized by shallow pockets and the absence of gingival bleeding.^3^ However, there are a variety of case definitions,^4^, ^5^, ^6^ and these definitions refer to an array of clinical signs and symptoms, such as probing pocket depth (PPD), clinical attachment loss (CAL) and bleeding on probing (BOP).^7^ Consequently, we assume that there is heterogeneity in the definitions of periodontal health. The definition of periodontal health should be consistent, facilitating comparison of clinical studies.^8^ Periodontal health was recently defined as the absence of clinically detected inflammation by the 2018 World Workshop of the European Federation of Periodontology (EFP) and the American Academy of Periodontology (AAP).^9^ This EFP/AAP definition is mainly based on PPD and BOP scores. To date, no overview of periodontal health definitions has been conducted. Therefore, this systematic review (SR) investigates the current scientific literature related to the definition of periodontal health.

## 2 MATERIALS AND METHODS

### 2.1 Protocol development

The protocol for this SR was developed “a priori,” following an initial discussion among members of the research team according to the Cochrane Handbook for Systematic Reviews of Interventions and the guidelines of PRISMA and MOOSE.

### 2.2 Search strategy

A structured literature search of the National Library of Medicine, Washington, DC (PubMed‐MEDLINE), and the Cochrane Central Register of Controlled Trials (Cochrane‐CENTRAL) was performed up to May 2019. Since the Centers for Disease Control and the American Academy of Periodontology (CDC/AAP) case definition of periodontal disease was updated in 2012, this report covers all studies published and cited since January 2013. We hand‐searched all of the reference lists of selected papers. This forward citation check was carried out in four rounds to identify additional published work that could meet the eligibility criteria of the study, so‐called “snowball procedure.” For details regarding the search terms used, see Appendix S1.

### 2.3 Eligibility criteria

Publications were included only when they (a) were original studies, (b) were conducted in a human population, (c) were published in English, (d) contained a defined group of periodontal health or a non‐defined control group as an opposite to the defined periodontal disease, and (e) their definitions described measurements and identified thresholds.

### 2.4 Screening and selection

Two reviewers (AL and RZT) screened the titles and abstracts of the studies obtained during the search for eligible papers independently. After the screening, the reviewers read the full texts of eligible papers in detail. Any disagreement concerning eligibility was resolved by consensus, and if conflict persisted, the decision was settled through arbitration led by a third reviewer (DES). The papers that met all the selection criteria were processed for data extraction.

### 2.5 Assessment of heterogeneity

Heterogeneity across studies was detailed according to the following factors: study design, published journal type, subject characteristics, potential confounding factors, measurement tools and procedures, the number of explicit definitions, clinical parameters and cut‐off values.

### 2.6 Methodological assessment of risk of bias

The two reviewers independently scored the methodological qualities of each study as well (AL and RZT). The appropriate critical appraisal checklists from the Joanna Briggs Institute were used depending on the study design of the paper.^10^ Studies that met 80% of the criteria were considered to have a low risk of bias. And 60% to 79% was a moderate one; 40% to 59% criteria were substantial one; and less than 40%, high one.^11^

### 2.7 Data extraction and analysis

The characteristics of the published journal type, study design, country, sample frame, sample size, group, age, gender, smoking status, medical condition, examination area, measurement tool, probing location and definition of periodontal health were extracted. Papers that included detailed measuring parameters and clear cut‐off values were regarded as having an “explicit definition”.^12^ Moreover, the “explicit definition” papers that used references to support their definitions were viewed as having a “valid definition”.^13^ The extracted criteria for periodontal health were recorded with Microsoft Excel 2017 (Microsoft). All quantitative analyses were conducted with SPSS Statistics 25 (SPSS Inc).

## 3 RESULTS

### 3.1 Search results

**FIGURE 1:**
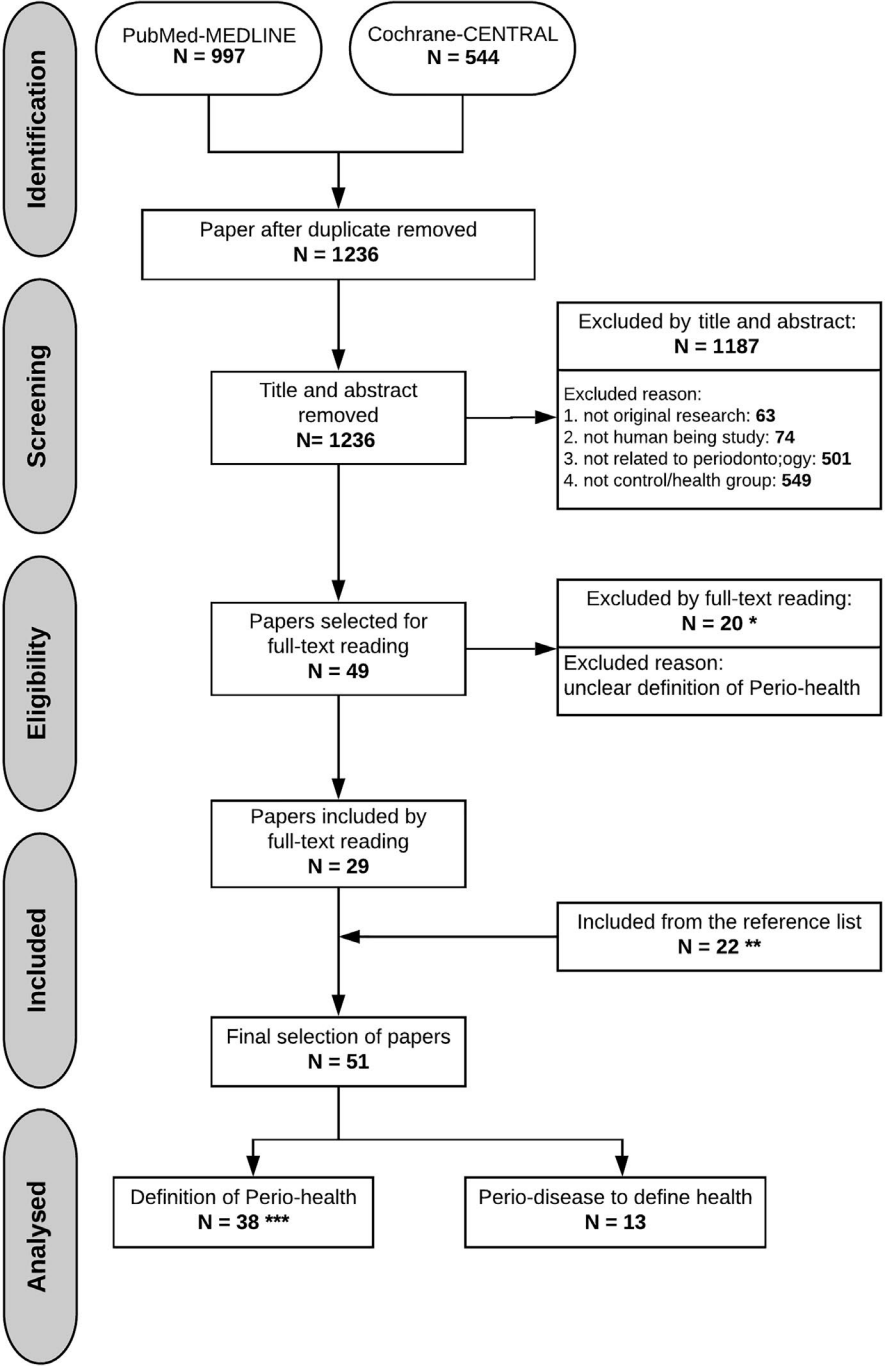
Flow of information through the different phases of the systematic review. *see Appendix S2, **see Appendices S3 and S4, *** see Table 3. Abbreviations: Perio‐health, periodontal health; Perio‐disease, periodontal disease.

The search through online databases resulted in 1236 unique studies (Figure 1). The initial screening of the titles and abstract resulted in 49 studies that went on to full‐text review. Then, a detailed reading of the full texts was performed. Two independent reviewers excluded 20 studies (Appendix S2), leaving 29 eligible papers. Furthermore, a manual search through the reference list of the 29 papers led reviewers to identify 22 additional relevant studies (Appendices S3 and S4). Finally, a total of 51 studies were included for the evaluation of the definition of periodontal health. Among the selected papers, 38 provided a definition of periodontal health. Thirteen studies did not report an explicit periodontal health definition and rather referenced periodontal health as the opposite of disease. This study outlines the characteristics of the included papers. These characteristics are summarized in Table 1.

**TABLE 1:** Overview of the studies processed for data extraction

Reference (year) Type of journal Risk of bias	Study design Sample frame Country	Sample size of all Healthy group: number/ age/ gender	Smoking status Medical condition	Examination area Measurement tool Probing location	Explicit definition of periodontal health or Opposite of periodontal disease
Mourão et al, 2013^34^ Medical journal Substantial	RCT study Dental clinic Brazil	ALL: (n = 60) Periodontal health group: (n = 20) 48.6 ± 7.4, ♀: 12/ ♂: 8	Not recorded Not recorded	Full mouth Probe type unclear Inter‐proximal sites	Explicit definition of periodontal health
Jones et al, 2013^35^ Dental journal Substantial	RCT study General population United Kingdom	ALL: (n = 369) 6‐month group: (n = 125) 37.1 ± 10.4, ♀: 68 (54.4%)/ ♂: 57 (45.6%) 12‐month group: (n = 122) 39.6 ± 10.8, ♀: 79 (64.8%)/ ♂: 43 (35.2%) 24‐month group: (n = 122) 36.4 ± 10.6, ♀: 88 (72.1%)/ ♂: 34 (27.9%)	Recorded Excluded	Full mouth WHO probe Six sites	Basic Periodontal Examination (BPE) Explicit definition of periodontal health
Graziani et al, 2018^36^ Periodontal journal Moderate	RCT study Dental hospital Italy	ALL: (n = 60) Group 1: (n = 15) 28.7 ± 9.8, ♀: 6 (40%)/ ♂: 9 (60%) Group 2: (n = 14) 26.1 ± 3.7, ♀: 8 (57%)/ ♂: 6 (43%) Group 3: (n = 16) 26.4 ± 5.2, ♀: 9 (56%)/ ♂: 7 (44%) Group 4: (n = 15) 26.4 ± 5.4, ♀: 8 (53%)/ ♂: 7 (47%)	Excluded Excluded	Full mouth UNC‐15 probe Six sites	Consensus report of the 5th European Workshop in periodontology^29^ © 2020 The Authors. International Journal of Dental Hygiene published by John Wiley & Sons Ltd Explicit definition of periodontal health
Sukhtankar et al, 2013^37^ Medical journal Moderate	Non‐randomized experimental study Department of periodontics and oral implantology India	© 2020 The Authors. International Journal of Dental Hygiene published by John Wiley & Sons Ltd (24‐55), ♀: 20/ ♂: 20 © 2020 The Authors. International Journal of Dental Hygiene published by John Wiley & Sons Ltd © 2020 The Authors. International Journal of Dental Hygiene published by John Wiley & Sons Ltd	Excluded Excluded	Full mouth UNC‐15 probe Site unclear	Explicit definition of periodontal health
Sharma et al, 2014 ^38^ Medical journal Moderate	Non‐randomized experimental study Dental hospital India	© 2020 The Authors. International Journal of Dental Hygiene published by John Wiley & Sons Ltd © 2020 The Authors. International Journal of Dental Hygiene published by John Wiley & Sons Ltd 25‐60	Excluded Excluded	Full mouth Probe type unclear Six sites	© 2020 The Authors. International Journal of Dental Hygiene published by John Wiley & Sons Ltd^39^: Opposite of periodontal disease
Guentsch et al, 2014^40^ Periodontal journal Moderate	Non‐randomized experimental study Dental hospital Germany	ALL: (n = 30) Periodontal health group: (n = 15) 26 (23‐39), ♀: 11/ ♂: 4	Excluded Excluded	Full mouth UNC‐15 probe Six sites	Armitage classification^17^: Explicit definition of periodontal health
Hassan et al, 2015^41^ Medical journal Moderate	Non‐randomized experimental study General population Egypt	ALL: (n = 30) Periodontal health group: (n = 10) 37.81 ± 8.3, ♀: 6/ ♂: 4	Excluded Excluded	Full mouth Michigan 0 probe Six sites	Explicit definition of periodontal health
Leite et al, 2014^42^ Medical journal Low	Non‐randomized experimental study General hospital Brazil	ALL: (n = 55) Periodontal health group: (n = 55) 33.18 ± 6.42, ♀: 67%/ ♂: 33%	Excluded Excluded	Full mouth Michigan 0 probe Four sites	Armitage classification^17^: Explicit definition of periodontal health
Al‐Hamoudi et al, 2018 ^43^ Dental journal Low	Non‐randomized experimental study Dental hospital Saudi Arabia	ALL: (n = 137) Obese patients without CP: (n = 34) 37.5 (31‐42), ♀: 2/ ♂: 32 Non‐obese patients without CP: (n = 33) 36.2 (33‐42), ♀: 0/ ♂: 33	Excluded Excluded	Full mouth UNC‐15 periodontal probe Six sites	Armitage classification^17^: Opposite of periodontal disease
Muthu et al, 2015^44^ Dental journal Moderate	Non‐randomized experimental study Dental hospital India	ALL: (n = 220) (35‐50), ♀: 96/ ♂: 124 Control group: (n = 90)	Excluded Excluded	Examination area unclear Probe type unclear Site unclear	Explicit definition of periodontal health
Raber‐Durlacher et al, 2013^45^ Medical journal High	Cohort study Dental hospital Netherlands	ALL: (n = 18) 41.8 ± 13.4 (19‐64) ♀: 11 (61%)/ ♂: 7 (39%) Periodontal health group: (n = 5)	Not recorded Not recorded	Full mouth Probe type unclear Four sites	Explicit definition of periodontal health
Ricardo et al, 2015 ^15^ Medical journal Moderate	Cohort study Population United States	ALL: (n = 10,755): 41.5 ± 0.5, ♀: 50%/ ♂: 50% CKD (+) without periodontitis group: (n = 1,142): 51.9 ± 1.2, ♀: 62.1%/ ♂: 37.9% CKD (‐) without periodontitis group: (n = 8,795): 39.6 ± 0.4, ♀: 49.8%/ ♂: 50.2%	Recorded Chronic kidney disease (CKD) patients	Full mouth Probe type unclear Site unclear	CDC/AAP case definition^6^: Opposite of periodontal disease
Lee et al, 2017 ^46^ Medical journal Substantial	Cohort General population Korea	ALL: (n = 354,850) Periodontal health group: (n = 154,824) 40‐49:46.8%, 50‐59:27.6%, 60‐69:19.6%, 70‐79:6%. ♀: 49.2%, ♂: 50.8%	Recorded Excluded	Full mouth Probe type unclear Inter‐proximal sites	Armitage classification^17^: Opposite of periodontal disease
Lourenço et al, 2014^18^ Periodontal journal Moderate	Case‐control study Division of Graduate Periodontics Brazil	ALL: (n = 97) Periodontal health group: (n = 27) 24.2 ± 6.9, ♀: 77.8%/ ♂: 22.2%	Recorded Excluded	Full mouth UNC‐15 probe Site unclear	Armitage classification^17^: Explicit definition of periodontal health
Zimmermann et al, 2013^47^ Periodontal journal Low	Cross‐sectional Dental hospital Brazil	ALL: (n = 78) NW non‐periodontitis (NP) group: (n = 20) 42.9 ± 7.2, ♀: 14/ ♂: 6 Obese non‐periodontitis group: (n = 18) 43.2 ± 7.4, ♀: 14/ ♂: 4	Excluded Excluded	Full mouth UNC‐15 probe Six sites	Armitage classification^17^: Explicit definition of periodontal health
Apatzidou et al, 2013^48^ Dental journal Moderate	Cross‐sectional Department of periodontology Greece	ALL: (n = 78) Healthy individuals: (n = 27) 31 ± 5, ♀: 15/ ♂: 12	Excluded Excluded	Full mouth UNC‐15 probe Six sites	Explicit definition of periodontal health
Ebersole et al, 2013^49^ Medical journal Low	Cross‐sectional Population United States	ALL: (n = 80) Healthy adults: (n = 30) 31.4 ± 6.8, ♀: 46.7%/ ♂: 53.3%	Recorded Excluded	Full mouth UNC‐15 probe Inter‐proximal sites	Armitage classification^17^: Explicit definition of periodontal health
Rathnayake et al, 2013^50^ Periodontal journal Low	Cross‐sectional Dental hospital Sweden	ALL: (n = 451) PD‐ group: (303) 42.6 ± 15.5, gender unclear	Recorded Recorded	Full mouth UNC‐15 probe Four sites	Explicit definition of periodontal health
Wang et al, 2013^51^ Medical journal High	Cross‐sectional Dental hospital China	ALL: (n = 16) 30‐65, gender unclear	Excluded Excluded	Examination area unclear Probe type unclear Site unclear	Explicit definition of periodontal health
Gursoy et al, 2013^52^ Periodontal journal Moderate	Cross‐sectional Population Finland	ALL: (n = 230) Control subject group: (n = 81) 47.9 ± 5.7, ♀: 64.2%/ ♂: 35.8%	Recorded Excluded	Full mouth Probe type unclear Four sites	Explicit definition of periodontal health
Kebschull et al, 2013^53^ Dental journal Substantial	Cross‐sectional Clinic of post‐doctoral periodontics United States	ALL: (n = 310) “Healthy” group: (n = 69) 45.7 ± 11.6 (24‐76), ♀: 50.8%/ ♂: 49.2%	Excluded Excluded	Full mouth Probe type unclear Six sites	Armitage classification^17^: Explicit definition of periodontal health
Salazar et al, 2013^54^ Periodontal journal Moderate	Cross‐sectional Population Germany	ALL: (n = 400) Healthy periodontium group: (n = 20) 48.6 ± 11.4, ♀: 50%/ ♂: 50%	Recorded Excluded	Examination area unclear SHIP‐2: PCP11 probe; SHIP‐TREND: PCPUNC probe 15 Site unclear	Explicit definition of periodontal health
Gokhale et al, 2013 ^55^ Medical journal Low	Cross‐sectional Department of Periodontics India	ALL: (n = 120) 30‐60 Periodontal health group: (n = 30)	Excluded Excluded	Full mouth UNC‐15 probe Four sites	Armitage classification^17^: Explicit definition of periodontal health
Wara‐aswapati et al, 2013 ^56^ Periodontal journal Moderate	Cross‐sectional General hospital Thailand	ALL: (n = 35) Control individuals without periodontitis: (n = 16) 34.0 ± 15.8, ♀: 14/ ♂: 2	Not recorded Excluded	Area unclear UNC‐15 probe Site unclear	Armitage classification^17^: Opposite of periodontal disease
Javed et al, 2014 ^57^ Periodontal journal Low	Cross‐sectional Dental hospital Pakistan	ALL: (n = 88) Controls: (n = 28) 51.7 ± 12.9, ♀: 0/ ♂: 28	Excluded Excluded	Full mouth Hu‐Friedy probe Six sites	Armitage classification^17^: Opposite of periodontal disease
Kim et al, 2013^58^ Dental journal Low	Cross‐sectional Dental hospital Korea	ALL: (n = 125) 57.85 ± 1.03, ♀: 48/ ♂: 77	Recorded Excluded	Area unclear WHO probe Six sites	WHO community periodontal index of treatment needs ^59^ Explicit definition of periodontal health
Pushparani et al, 2014^60^ Periodontal journal Low	Cross‐sectional Department of periodontology India	ALL: (n = 600) Control healthy individual: (n = 150) 35.46 ± 610.74, ♀: 70/ ♂: 80 Type 2DM without periodontitis: (n = 150) 46.26 ± 10.02, ♀: 72/ ♂: 78	Excluded Excluded	Area unclear Probe type unclear Site unclear	Armitage classification^17^: Opposite of periodontal disease
Shetty et al, 2016 ^61^ Medical journal moderate	Cross‐sectional Dental hospital India	ALL: (n = 120) Healthy group: (n = 30)	Excluded Excluded	Area unclear Probe type unclear Site unclear	Explicit definition of periodontal health
Panezai et al, 2018 ^62^ Medical journal Low	Cross‐sectional Dental hospital Pakistan	ALL: (n = 86) Healthy group: (n = 14) 44.4 ± 6.6, ♀: 5/ ♂: 9	Recorded Excluded	Full mouth Hu‐Friedy probe Four sites	Explicit definition of periodontal health
Huang et al, 2018^63^ Medical journal Moderate	Cross‐sectional Dental hospital China	ALL: (n = 68) ♀: 31 (43 ± 12.1)/ ♂: 37 (47 ± 11.7) Healthy group: (n = 20)	Not recorded Excluded	Area unclear Probe type unclear Site unclear	Armitage classification^17^: Explicit definition of periodontal health
Papathanasiou et al, 2014^64^ Periodontal journal Moderate	Cross‐sectional Population United States	ALL: (n = 42) periodontally healthy group: (n = 14) 26.3 ± 2.6, ♀: 78.6%/ ♂: 21.4%	Excluded Excluded	Full mouth UNC‐15 probe Six sites	Explicit definition of periodontal health
Mesa et al, 2014^65^ Periodontal journal Low	Cross‐sectional Dental hospital Spain	ALL: (n = 77) Periodontal health group: (n = 36) 46.25 (19‐79), ♀: 46/ ♂: 31	Recorded Excluded	Full mouth UNC‐15 probe Six sites	Explicit definition of periodontal health
Schjetlein et al, 2014^14^ Medical journal Moderate	Cross‐sectional study General hospital Diabetes patients Denmark	ALL: (n = 62) 57.0 (51‐60), ♀: 28/ ♂: 34 Without periodontitis group: (n = 49) 57.0 (51‐61), ♀: 24/ ♂: 25	Recorded Excluded	Full mouth WHO probe Site unclear	Periodontal Screening Index Explicit definition of periodontal health
Ramírez et al, 2014^66^ Dental journal Low	Cross‐sectional General hospital Colombia	ALL: (n = 44) Periodontal health group: (n = 22) 40.6 ± 8.6, ♀: 17 (77.3%)/ ♂: 5 (22.7%)	Recorded Excluded	Full mouth Probe type unclear Site unclear	Armitage classification^17^: Explicit definition of periodontal health
Beklen and Tsaous Memet, 2014 ^67^ Medical journal Substantial	Cross‐sectional General hospital Turkey	ALL: (n = 20) Periodontal health group: (n = 10) 33‐39, gender unclear	Excluded Excluded	Full mouth Probe type unclear Inter‐proximal sites	Explicit definition of periodontal health
Singh et al, 2014^68^ Periodontal journal Low	Cross‐sectional Department of periodontics and oral implantology India	ALL: (n = 106) Periodontally healthy individuals: (n = 22) 27.5 (22‐50), ♀: 16/ ♂: 6	Excluded Excluded	Full mouth Probe type unclear Six sites	Explicit definition of periodontal health
Duran‐Pinedo et al, 2014^69^ Medical journal Substantial	Cross‐sectional General hospital United States	ALL: (n = 13) Periodontally healthy individuals: (n = 6) Age unclear, gender unclear	Excluded Excluded	Examination area unclear Probe type unclear Site unclear	Explicit definition of periodontal health
Tabari et al, 2013^70^ Dental journal Moderate	Cross‐sectional Department of Periodontology Iran	ALL: (n = 50) Individuals with a healthy periodontium: (n = 25) 20‐45, ♀: 11 (44%)/ ♂: 14 (56%)	Excluded Excluded	Full mouth UNC‐15 probe Four sites	Armitage classification^17^: Explicit definition of periodontal health
Tabari et al, 2013^70^ Periodontal journal Low	Cross‐sectional Department of Periodontology Iran	ALL: (n = 40) Periodontally healthy individuals: (n = 20) 33.85 ± 6.84, ♀: 65%/ ♂: 35%	Excluded Excluded	Full mouth UNC‐15 probe Four sites	Armitage classification^17^: Explicit definition of periodontal health
Garneata et al, 2015^16^ Medical journal Moderate	Cross‐sectional study General hospital Romania	ALL: (n = 238) 57.0 (50.0‐64.8), ♀: 40%/ ♂: 60% Periodontal health group: (n = 58) 55.5 (42.3‐61.0), ♀: 43%/ ♂: 57%	Recorded Stable chronic hemodialysis patients	Full mouth Probe type unclear Site unclear	Explicit definition of periodontal health
Torrungruang et al, 2015 ^71^ Medical journal Substantial	Cross‐sectional study Population Thailand	ALL: (n = 1,362) No/mild periodontitis: (n = 479) 46.6 ± 4.4, ♀: 211/ ♂: 268	Recorded Not recorded	Full mouth Probe type unclear Six sites	CDC/AAP case definition^6^: Opposite of periodontal disease
Ghallab et al, 2015^72^ Periodontal journal Moderate	Cross‐sectional Dental hospital Egypt	ALL: (n = 50) Periodontal health group: (n = 10) 47.8 ± 2.9, ♀: 5/ ♂: 5	Excluded Excluded	Full mouth Michigan 0 probe Six sites	Armitage classification^17^: Explicit definition of periodontal health
Lavu et al, 2015^73^ Medical journal Moderate	Cross‐sectional Dental hospital India	ALL: (n = 400) Periodontal health group: (n = 200) 29.64 ± 5.5 (20‐55), ♀: 52.4%/ ♂: 47.5%	Excluded Excluded	Full mouth UNC‐15 probe Six sites	Armitage classification^17^: Explicit definition of periodontal health
Kurşunlu et al, 2015^74^ Dental journal Moderate	Cross‐sectional study Department of periodontology Turkey	ALL: (n = 80) Periodontally healthy subjects (n = 20)	Excluded Excluded	Full mouth Probe type unclear Site unclear	Armitage classification^17^: Explicit definition of periodontal health
Chaiyarit et al, 2015^75^ Dental journal High	Cross‐sectional General hospital Thailand	ALL: (n = 90) Healthy subjects: (n = 30) 54.4 ± 11.03 (35‐75), ♀: 17/ ♂: 13	Not recorded Excluded	Examination area unclear Probe type unclear Site unclear	Armitage classification^17^: Explicit definition of periodontal health
Özcan et al, 2015^76^ Dental journal Moderate	Cross‐sectional Department of periodontology Turkey	ALL: (n = 72) Healthy subjects: (n = 23) 34.50 ± 7.09 (35‐75), ♀: 11/ ♂: 12	Excluded Excluded	Examination area unclear Michigan 0 probe Site unclear	Explicit definition of periodontal health
Kirst et al, 2015^77^ Medical journal Substantial	Cross‐sectional General hospital United States	ALL: (n = 50) Healthy controls: (n = 25)	Not recorded Excluded	Examination area unclear Probe type unclear Site unclear	Explicit definition of periodontal health
Velosa‐Porras et al, 2016 ^78^ Dental journal Low	Cross‐sectional Dental hospital Colombia	ALL: (n = 150) Mean = 50.2 Periodontal health group: (n = 75) ♀: 44/♂: 31	Recorded Excluded	Full mouth Electronic probe Site unclear	Armitage classification^17^: Opposite of periodontal disease
Prodan et al, 2016^79^ Medical journal Moderate	Cross‐sectional Population Netherlands	ALL: (n = 261) 22.6 (18.0,32.0) ♀: 116/ ♂: 145	Excluded Excluded Student of university	Full mouth Probe type unclear Site unclear	Dutch periodontal screening index (DPSI) ^80^ Explicit definition of periodontal health
Noguera‐Julian et al, 2017 ^81^ Medical journal Moderate	Cross‐sectional Dental hospital United States	ALL: (n = 50) 45.3 (37.0‐53.0) ♀: 17/ ♂: 32/ Trans: 1	Recorded Excluded	Full mouth Probe type unclear Inter‐proximal sites	CDC/AAP case definition^6^: Opposite of periodontal disease
Sağlam et al, 2017^82^ Dental journal Moderate	Cross‐sectional Dental hospital Turkey	ALL: (n = 60) ♀: 33/ ♂: 27 Periodontal health group: (n = 20) 30.62 ± 7.65	Excluded Excluded	Full mouth Probe type unclear Six sites	Explicit definition of periodontal health

### 3.2 Methodological quality assessment

The methodological quality of the included studies was used to estimate the potential risk of bias and is presented in detail in Appendices S5.1‐5. The estimated potential risk of bias was low for 15 studies, moderate for 25 studies, substantial for eight studies, and high for three studies.

### 3.3 Study characteristics

The papers were published in journals of different categories, targeting periodontology (29%), dentistry (29%) and general medicine (41%). The studies were designed as cross‐sectional studies (37/51), longitudinal studies (4/51), and randomized or non‐randomized allocated control studies (10/51). A total number of 372 983 individuals were enrolled in the studies, ranging from 18 to 354 850 individuals for each one (mean: 7313, SD: 49 660, median: 78). Most publications were authored by research groups in India (14%) and the United States (12%).

Most studies (82%) recruited patients from a hospital setting with comorbidities such as diabetes,^14^ chronic kidney disease^15^ or chronic haemodialysis.^16^ Concerning confounding factors, such as smoking habits and medical condition, 28 of the studies excluded participants with smoking habits. Those with complicated medical conditions were excluded from 45 studies (Table 1).

### 3.4 Measurement methods

In 39 out of the 51 papers, a full‐mouth assessment was conducted (Table 1). Various types of periodontal probes were used. Twenty‐four studies did not report the details of the probe, 16 studies used the UNC‐15 probe, and four studies used the Michigan 0 probe. The number and location of probing sites varied. Four sites (mesiobuccal, mesiolingual, distobuccal and distolingual) per tooth were used in 8 studies, and six sites (mesiobuccal, midbuccal, distobuccal, mesiolingual, midlingual and distolingual) per tooth were used in 17 studies. Moreover, five studies specifically measured the indicators at the location of the inter‐proximal sites.

### 3.5 Presence of an explicit or valid definition according to journal type, study design and risk of bias

A precise definition of periodontal health is offered in 38 (75%) of the included studies. The remaining 13 papers provided the references and defined the opposite of disease as periodontal health (Table 2). An explicit definition with a supporting reference was reported in 19 papers. In contrast, the other 19 studies only used a definition rather than indicating any reference (Table 2; for details, see Appendix S6). The two most frequently used references were the Armitage classification (1999),^17^ used in 22 papers, and the CDC/AAP case definition,^6^ used in five papers. None of the papers reporting details of the classification followed the original proposed definition strictly, but a wide variance was applied (Appendices S7.1‐2).

**TABLE 2:** Classification of included papers according to explicit and valid definitions

ALL = 51	Full‐text reading	N (%)		Definition analysing	N (%)
1	Definition of health^a^	38 (74.5)	1a	Definition of health with reference^b^	19 (37.25)
			1b	Definition of health without reference	19 (37.25)
2	Disease to define health	13 (24.5)	2a	Definition of disease with reference	12 (23.5)
			2b	Definition of disease without reference	1 (2)

^a^ The “only definition” and “reference and definition” groups were regarded as explicit definitions of periodontal health.

^b^ The “reference and definition” group was regarded as a valid definition of periodontal health.

The number of explicit and valid periodontal health definitions was sub‐analysed according to journal categories, study designs and resource of patients as well as assessed methodological risk of bias. In the periodontal journals, the definitions used were more explicit (87%) than those used in the dental or medical journals (Appendix S8.1). Moreover, the papers collected from a department of periodontology tended to provide explicit definitions (91%) compared with other studies. The studies scoring a low risk of bias for the methodical quality had more valid definitions (Appendix S8.2).

### 3.6 Clinical parameters and cut‐off values

Table 3 summarizes the different periodontal health definitions used (38 studies). Notably, Loureço provided two definitions of periodontal health in the one study.^18^ Therefore, the table contains 39 definitions. The table also presents the differences regarding cut‐off points, PPD, CAL, BOP, and other relevant information for each study. Probing pocket depth was almost used for all definitions (n = 35), whereas BOP was used in less than half of the cases (n = 16). Probing pocket depth appeared in nine studies used as a single criterion. A combination of PPD with CAL appeared in 10 studies. A combination of PPD with BOP appeared in five studies, and a triple set of PPD, CAL, and BOP was used in 10 papers.

**TABLE 3:** Summary of periodontal health definitions

Papers (N)/definitions (n)	PPD (mm)	CAL (mm)	BOP (%)	Other	Reference
N = 38/ n = 39	n = 35	n = 24	n = 16		
1	<3				Beklen et al, 2014^67^
2	<3				Özcan et al, 2015^76^
3	<3				Duran‐Pinedo et al, 2014^69^
4	<3.5				Jones et al, 2013^35^
5	<3.5				Schjetlein et al, 2014^14^
6	<4				Garneata et al, 2015^16^
7	<4				Gursoy et al, 2013^52^
8	<4			No clinical sign + no X‐ray bone loss	Ramírez et al, 2014^66^
9	<5				Prodan et al, 2016^79^
10		<3			Kirst et al, 2015^77^
11		<3			Kim et al, 2013^58^
12		<3			Graziani et al, 2018^36^
13				No X‐ray bone loss	Rathnayake et al, 2013^50^
14	=0	=0		No clinical sign + no X‐ray bone loss	Huang et al, 2018^63^
15	<3	<3			Wang et al, 2013^51^
16	<3	=0			Guentsch et al, 2014^40^
17	<3	=0			Tabari et al, 2014^70^
18	<3	=0			Pushparani et al, 2014^60^
19	<3	=0		GI = 0 + PI = 0	Ghallab et al, 2015^72^
20	<3	=0		GI = 0 + PI = 0	Hassan et al, 2015^41^
21	<3	<2			Mesa et al, 2014^65^
22	<3	<3			Zimmermann et al, 2013^47^
23	<4	<4			Kebschull et al, 2013^53^
24	<3		<10		Tabari et al, 2013^70^
25	<3		<10		Apatzidou et al, 2013^48^
26	<3		<30		Salazar et al, 2013^54^
27	<4		<15		Muthu et al, 2015^44^
28	<4		<10		Raber‐Durlacher et al, 2013^45^
29	<3	=0	=0		Kurşunlu et al, 2015^74^
30	<3	<3	=0		Mourão et al, 2013^34^
31	<3	=0	=0	No clinical sign + no history	Lavu et al, 2015^73^
32	<3	=0	<10		Singh et al, 2014^68^
33	<3	<1	<10		Sukhtankar et al, 2013^37^
34	<3	<2	<20	No X‐ray bone loss	Sağlam et al, 2017^82^
35^a^	<3	<3	<10		Lourenço et al, 2014^18^
	<4	<4	<5		Lourenço et al, 2014^18^
36	<3	<3	<10	No X‐ray bone loss	Leite et al, 2014^42^
37	<3	≤3	<20		Papathanasiou et al, 2014^64^
38	<4	<2	<10		Ebersole et al, 2013^49^

Abbreviations: BOP, bleeding on probing; CAL, clinical attachment level; PDD, probing pocket depth.

^a^ Lourenço et al provided two sets of periodontally healthy definition in one paper. 'Gray shades' means a single criterion or combination involving PPD/CAL/BOP to define periodontal health.

**FIGURE 2:**
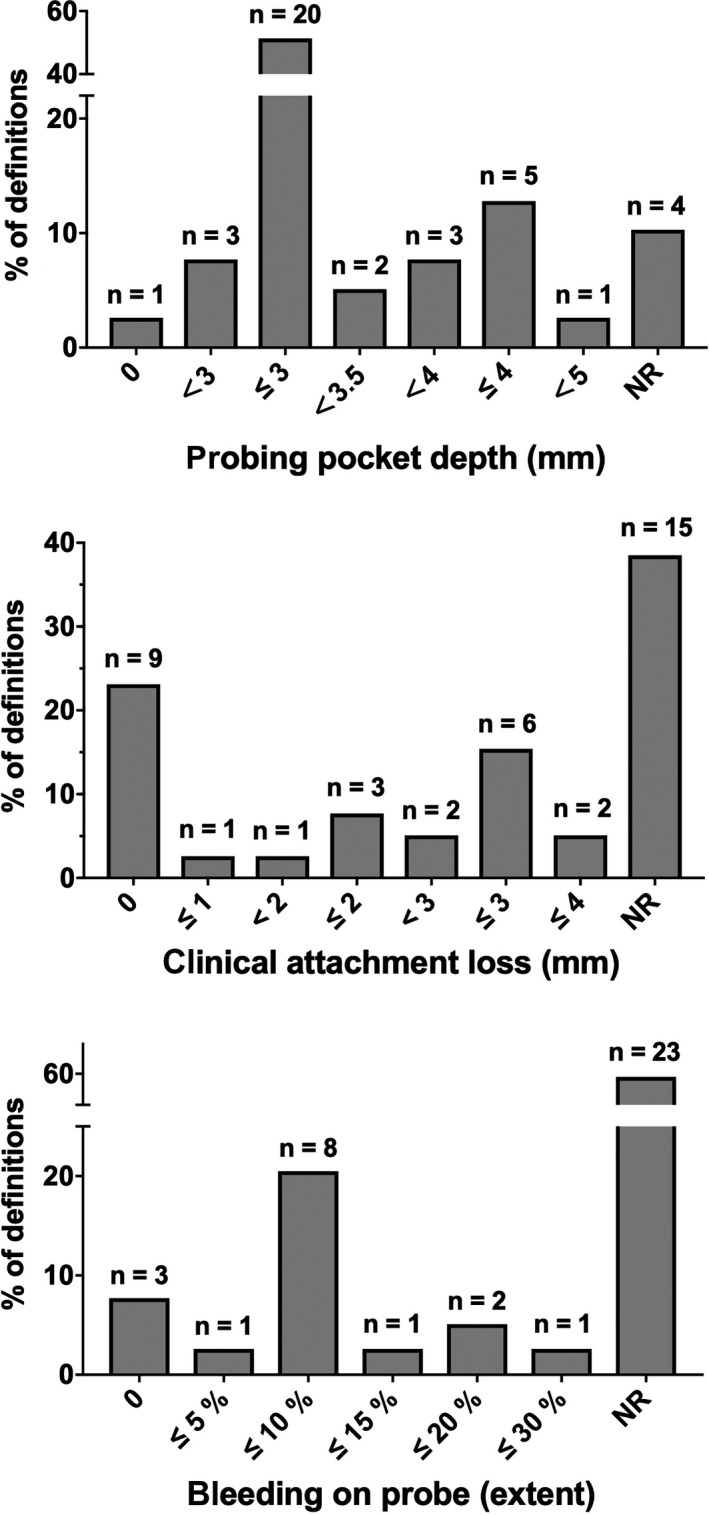
Distribution of severity and extent for PPD (A), CAL (B) and BOP (C) used to define periodontal health among 36 studies showed by number and percentage. Notably, one of studies provided two definitions of periodontal health. Therefore, the total number of definitions is 37

Figure 2A‐C presents the numbers of papers using a threshold. The most frequently used PPD cut‐off was ≤3 mm, which appeared in 20 studies. However, 11 studies reported a threshold of 3.5 mm or higher. A considerable amount of variety was observed concerning the CAL threshold, ranging from 0 to 4 mm. Nine studies reported the absence of CAL, and 15 studies did not report CAL (Figure 2B). Figure 2C demonstrates that among the reported BOP thresholds, the most commonly used was 10% sites, but the majority of the included papers (n = 23) did not report BOP.

## 4 DISCUSSION

This SR aims to conduct an exploratory analysis of the definitions of periodontal health and the methods used to measure a healthy periodontium. To the best of our knowledge, this is the first SR exclusively dedicated to exploring a variety of periodontal health definitions. Although the significance of periodontal health is well known, a universal, formal definition did not exist until the World Workshop on the Classification of Periodontal and Peri‐Implant Diseases and Conditions, which was organized by the EFP and the AAP.^9^ The main findings of this review were that (a) there is a lack of an explicit definition of periodontal health and consequently, a lack of application of references, (b) there is significant heterogeneity in measuring methods, and (c) there are considerable inconsistencies in the different periodontal parameters and cut‐off values used.

Operational definitions and consistent criteria for a healthy periodontium were not provided in the majority of papers. The studies that did not provide a definition or a reference were excluded (Appendix S2). Some did provide a definition but lacked a reference, and some only gave a reference but lacked a definition. Only 37% (19 of 51) of the papers included in this study reported an explicit definition with detailed clinical parameters and cited a reference. The two most commonly cited references were the 1999 International Workshop for the Classification of Periodontal Disease^17^ (21 out of 31) and the CDC/AAP case definition for population‐based studies of periodontitis^6^ (5 out of 31) (Appendix S6). Even when a proper reference was used, there existed a variety of interpretations. Misuse of the original criteria of the references created even more heterogeneity and introduces inevitable bias. As with the definition of periodontitis, it was difficult to achieve the goal of reproducing and analysing the results from different studies.^7^

A periodontal pocket is the most common sign of periodontitis and easy to detect and assess in the clinical practice using various periodontal probes. The regularization of using periodontal probes will raise the accuracy of the process of diagnosing the condition and evaluating the treatment outcome.^19^, ^20^ The present SR has identified a great amount of variety in the methods and materials used, such as the periodontal probing methods, particularly the type of probe and probing site. The procedure of measuring PPD and CAL was described as being assessed by either four or six sites per tooth. The number of sites used and especially the proportion of interdental sites assessed may influence the outcome. In any case, uniformity in material and methods can reduce the measurement bias. The EFP/AAP workshop recommended the use of an International Organization for Standardization (ISO) periodontal probe.^9^

A cut‐off or a reference point is needed to distinguish health from recurring signs and symptoms of periodontal disease.^8^ A wide range of parameters and cut‐offs were identified in the present systematic review. Probing pocket depth was the most frequently used periodontal parameter. Given the fact that it is rather easy to detect and measure, PPD has been recognized for many years as the essential parameter for the diagnosis of periodontal health and disease.^21^ Half of the studies (51%) reported a threshold of PPD ≤3 mm. This cut‐off value is also used to identify periodontal case types of health.^22^ In contrast, there were still 11 (29%) definitions that used the threshold of 3.5 mm PPD or deeper. The cut‐off PPD ≤3 mm might be excessively strict if a population is assessed such that only a few end up in the category of healthy. This may be the reason that researchers in large epidemiology studies stretch the PPD cut‐off point. For instance, Hugoson used the following cut‐off of periodontal health and disease: ≤10% sites with PPD ≥4 mm.^23^, ^24^ Nevertheless, even the largest cut‐off value of PPD did not exceed 5 mm in the current review. A systematic review reported that probing depth up to 6 mm or even more should be taken into account as a high‐risk factor to predict further disease progression in periodontal patients.^25^

Other frequently used parameters are CAL and BOP. Clinical attachment loss, the second most frequently used parameter, varies across studies. This was used in three (8%) studies as the single parameter and in 21 (55%) as an adjunct to PPD. The most commonly used threshold using CAL is the absence of attachment loss. As ageing comes with natural bone loss, some CAL is physiological. Therefore, the absence of CAL is likely due to the outdated concept. Periodontal health is identified as the absence of any deficit of supporting tissues.^8^ The strict and sometimes idealistic definition of absence of CAL can result in an overestimation of disease. The third most commonly used parameter, BOP, is never used alone, but serves as an adjunct. Notably, criteria consisting of BOP and PPD appeared in five (13%) articles, whereas BOP was only used in 16 out of 39 definitions (41%). The most frequently used BOP cut‐off is less than 10%. Stable periodontium can manifest as the absence of extensive BOP.^26^ The cut‐off values of BOP used to identify health and disease vary. A large‐scale epidemiological study used a cut‐off of <20% BOP,^23^ without referencing evidence. Patients with BOP sites ≥16% have a higher chance of losing attachment.^27^ After active non‐surgical treatment during the maintenance/supportive phase, the risk of tooth loss is considerably greater for patients with 30% bleeding.^28^ Overall, a limited amount of positive symptoms for BOP is accepted in the healthy periodontium. Interestingly, the most frequently used cut‐off value (BOP <10%) is consistent with the EFP/AAP classification. Nevertheless, there is no clear evidence to support the used cut‐off values. Compared to previous values, bleeding sites of 10% might underestimate the number of people with a healthy periodontium.

The current review is not without limitations. After a full‐text reading and analysis of the reference lists, 22 extra papers were included (for details, see Figure 1). Although this snowball procedure was conducted carefully, it remains possible that some studies describing periodontal health were not included in our search. Searching for definitions of periodontal health is complicated as it is often used as a category describing the opposite of disease. Thus, periodontal health often does not appear as a search term in the title and abstracts of studies. This also explains why the snowball procedure reveals more papers than those obtained from the initial search and selected based on the given criteria. A recommendation for further studies is that there is a need for evaluations such as what probe to use and what measurements to collect, in order to make a proper diagnosis for daily clinical practice and epidemiological studies.

The definition of periodontal health recommended by the EFP/AAP Workshop was defined as less than 10% of sites having BOP and PPD ≤3 mm in intact periodontium or ≤4 mm in reduced periodontium.^9^ Previous studies took CAL into account as a critical factor in describing accumulated lesions and the susceptibility of the disease.^29^, ^30^ However, loss of periodontal attachment has not been incorporated, partly because the newly proposed definition focuses on the current status of different periodontium. Periodontal inflammatory activity or inactivity can be identified according to the extent of BOP and PPD instead of CAL. Similar to the assessment of periodontal inflammatory burden,^31^ non‐bleeding pockets are regarded as periodontal tissue without inflammation. The quantity of inflammation is related to the inflamed periodontal surface area, which is calculated by the PPD values of bleeding teeth.

Periodontal health can also present in an anatomically reduced periodontium.^1^ In other words, periodontal health does not merely mean that there is an absence of supporting tissue deficit. It also refers to an individual's level of comfort, the stability of a functioning periodontium, and one's psychological and social well‐being. This concept of holistic periodontal health has not been taken into account in this paper. Notably, the feasibility of directly regarding the definition of periodontal health in a reduced periodontium (PPD ≤4 mm and BOP ≤10%) as the treatment goal among patients remains uncertain. However, a certain PPD value after treatment needs to be interpreted in the light of variance in susceptibility and personalized medicine. Lang and Tonetti built a functional diagram to assess periodontal risk in supportive periodontal therapy, which can help clinicians distinguish whether a treatment goal is reached or not.^32^ Moreover, the number of residual pockets with a probing depth of ≥5 mm to a certain extent reflects the degree of success of periodontal treatment, which is different from the PPD threshold in the new definition. In the randomized clinical trial,^33^ the subjects presenting ≤4 sites with PD ≥5 mm at one year represented a successful treatment outcome. Therefore, the endpoint of therapy should seek the most optimal balance between over‐ and underestimation of health status among treated periodontal patients. It is important to acknowledge the distinction between the diagnoses of periodontal health of initial patients versus treated patients. For the latter, a more flexible, comprehensive and detailed assessment would be recommended.

## 5 CONCLUSION

This SR revealed a variety of definitions of periodontal health in existing scientific literature. This heterogeneity was measured according to study characteristics, measurement methods, explicit definitions, references and cut‐off values used. The definition of periodontal health proposed by the EFP/AAP Workshop offers an opportunity for the field to standardize and achieve uniformity in terms of methodologies in order to draw comparisons between different studies. This study also revealed that the number of people thought to have periodontal disease is likely overestimated due to the strict cut‐off value.

## 6 CLINICAL RELEVANCE

### 6.1 Scientific rationale for the study

There is no standard reference for periodontal health, and the diagnostic properties of the various definitions have not been studied.

### 6.2 Principal finding

Marked heterogeneity in the definitions of different measuring methods and clinical parameters in periodontal health may be affecting interpretations of research.

### 6.3 Practical implications

The new definition of periodontal health proposed by the EFP/AAP workshop in 2018 offers an opportunity to standardize and unify the cut‐off values of clinical parameters, which would allow for a better comparison of clinical studies and support research and decision‐making.

## CONFLICT OF INTEREST

The authors declare that they have no conflicts of interest.

## AUTHOR CONTRIBUTIONS

An Li, first author, contributed to the acquisition, analysis and interpretation of data, and drafted the manuscript. Renske Z. Thomas, overall daily supervisor, contributed to the design of study, the acquisition, analysis and interpretation of data, and drafted the manuscript. Luc van der Sluis contributed to the design of study and critically revised the manuscript. Geerten‐Has Tjakkes contributed to the design and critically revised the manuscript. Dagmar Else Slot contributed to the conception and design of the study, supported the analysis and interpretation of the data, and critically revised the manuscript. All authors gave final approval and agreed to be accountable for all aspects of this work, ensuring its integrity and accuracy.

## Supporting information

Appendix S1‐S9
