# Supplementary material for: Definitions used for a healthy periodontium—A systematic review
Source: Int J Dent Hyg. 2020 May 24;18(4):327–43. doi: 10.1111/idh.12438 (PMC7687205; doi:10.1111/idh.12438)
Supplement: Supplementary file 1 — Appendix S1‐S9 [file IDH-18-327-s001.docx]

**Online Appendices**

**Appendices S1**

Search Strategy

**Appendices S2**

Table of reasons for excluding studies in the process of full-text reading (N = 25)

**Appendices S3**

Flow chart of hand-searching the reference lists of selected papers

**Appendices S4**

Table of eligible papers and their source (N = 22)

**Appendices S5**

Joanna Briggs Institute Critical Appraisal Checklist

**Appendices S6**

Classification tree of final selection of papers

**Appendices S7**

Definitions of periodontal health cited by Armitage (1999) classification and CDC/AAP case definition.

**Appendices S8**

Rate of explicit and valid periodontal health stratified by journal category, study design, risk of bias, and patient resource.

**Appendices S9**

References

**Appendices S1 Search Strategy**

*Box 1.* Search Strategy

Search terms used for Pub PubMed-MEDLINE and Cochrane-CENTRAL. The search strategy was customized according to the databases being used.

| The following strategy was used for the search. |
| --- |
| < [MeSH terms] Periodontal Diseases OR Periodontitis OR periodontal disease OR periodontal diseas* > |
| AND |
| < [text words] Healthy OR health OR Control > |

**Appendices S2 Table of reasons for excluding studies in the process of full-text reading (N = 25)**

| **Authors and published year** | **Reason for exclusion** |
| --- | --- |
| (Clarkson et al., 2013) | no real clinical trial but trial plan |
| (Vavricka et al., 2013) | control group compared to IBD, not periodontal control group |
| (Fernandes et al., 2017) | without periodontal control group information (no reference) |
| (Burgess et al., 2013) | without periodontal control group information (no reference) |
| (Biju et al., 2014) | without periodontal control group information (no reference) |
| (Gong et al., 2014) | without periodontal control group information (no reference) |
| (Novakovic et al., 2014) | without periodontal control group information (no reference) |
| (Paknejad et al., 2016) | without periodontal control group information (no reference) |
| (Toregeani et al., 2016) | without periodontal control group information (no reference) |
| (Herman et al., 2016) | without periodontal control group information (no reference) |
| (Konig et al., 2016) | without periodontal control group information (no reference) |
| (Saliasi et al., 2018) | without periodontal control group information (no reference) |
| (Valor et al., 2018) | without periodontal control group information (no reference) |
| (Hsu et al., 2015) | ICD-9-CM without detailed definition |
| (Chou et al., 2015) | ICD-9-CM without detailed definition |
| (Chen et al., 2015) | ICD-9-CM without detailed definition |
| (Huang et al., 2016) | ICD-9-CM without detailed definition |
| (Chung et al., 2016) | ICD-9-CM without detailed definition |
| (Pesevska et al., 2017) | position paper without detailed definition |
| (Mdala et al., 2014) | Prediction model, no epidemiologic study |
| (Laine et al., 2013) * | Prediction model, not epidemiologic study |
| (Loozen et al., 2014) * | without periodontal control group information (no reference) |
| (Abusleme et al., 2013) * | without periodontal control group information (no reference) |
| (Belstrom et al., 2014) * | without periodontal control group information (no reference) |
| (Kongstad et al., 2013) * | without periodontal control group information (no reference) |

* the five paper were from the reference list of 29 included articles after full-text reading.

**Appendices S3 Flow chart of hand-searching the reference lists of selected papers**

**
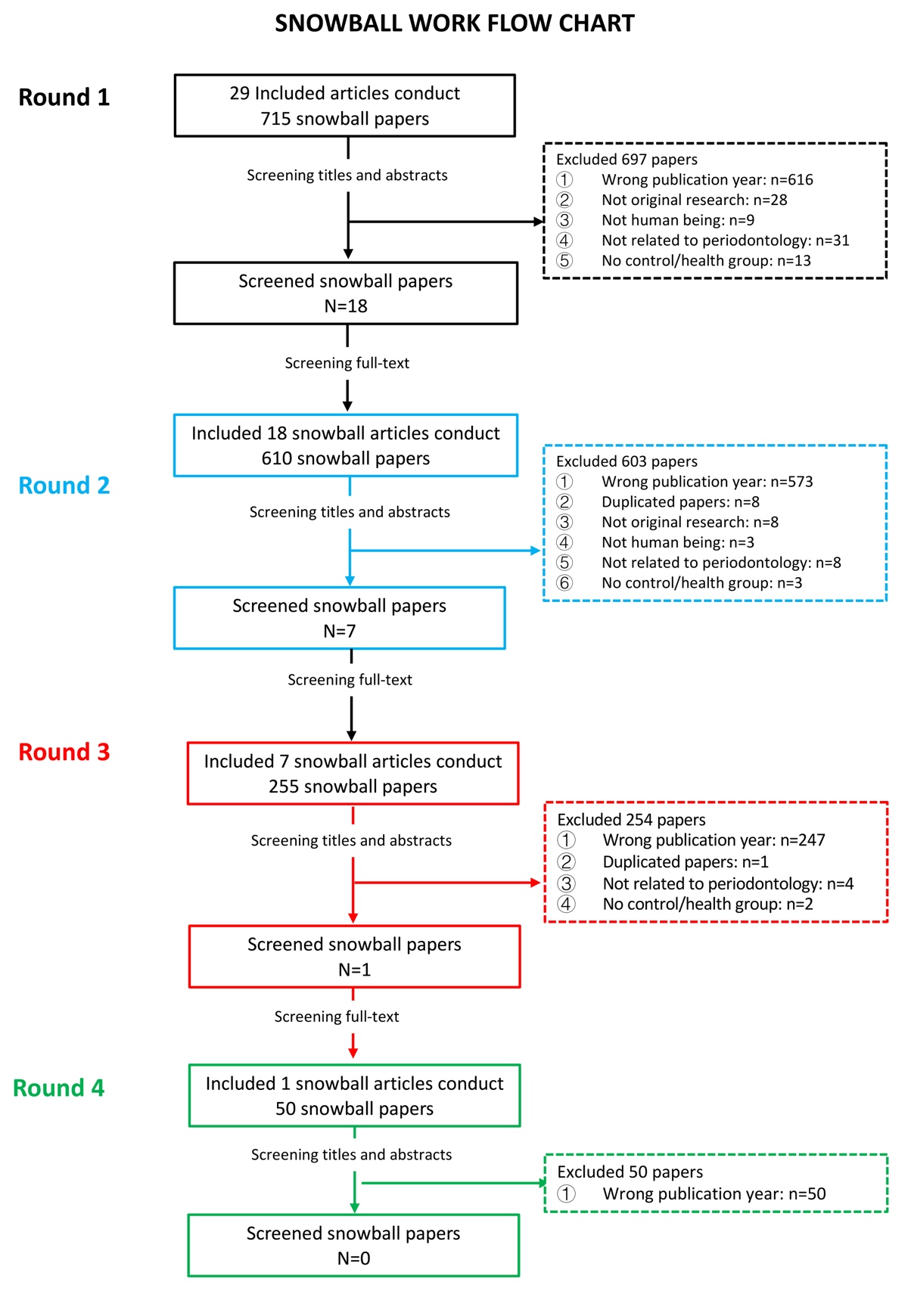
**

* snow ball means the further search work after full-text screening. We searched the eligible papers from the reference lists of the included papers.

**Appendices S4** Table of eligible papers and their source (N = 22)

| **Round** | **Authors and published year** | **Source of reference** |
| --- | --- | --- |
| 1 | (Chaiyarit et al., 2015) | (Prodan et al., 2016) |
| 1 | (Tabari et al., 2014) | (Ghallab et al., 2015) |
| 1 | (Ozcan et al., 2015) | (Ghallab et al., 2015) |
| 1 | (Zimmermann et al., 2013) | (Ghallab et al., 2015) |
| 1 | (Duran-Pinedo et al., 2014) | (Noguera-Julian et al., 2017) |
| 1 | (Kirst et al., 2015) | (Noguera-Julian et al., 2017) |
| 1 | (Kursunlu et al., 2015) | (Saglam et al., 2017) |
| 1 | (Beklen and Tsaous Memet, 2014) | (Saglam et al., 2017) |
| 1 | (Papathanasiou et al., 2014) | (Saglam et al., 2017) |
| 1 | (Apatzidou et al., 2013) | (Guentsch et al., 2014) |
| 1 | (Tabari et al., 2013) | (Hassan et al., 2015) |
| 1 | (Muthu et al., 2015) | (Al-Hamoudi et al., 2018) |
| 1 | (Wara-aswapati et al., 2013) | (Huang et al., 2018) |
| 1 | (Javed et al., 2014) | (Al-Hamoudi et al., 2018) |
| 1 | (Pushparani et al., 2014) | (Shetty et al., 2016a) |
| 1 | (Kim et al., 2013) | (Shetty et al., 2016a) |
| 2 | (Kebschull et al., 2013) | (Kursunlu et al., 2015) |
| 2 | (Salazar et al., 2013) | (Chaiyarit et al., 2015) |
| 2 | (Wang et al., 2013) | (Kirst et al., 2015) |
| 2 | (Rathnayake et al., 2013) | (Belstrom et al., 2014) |
| 2 | (Ebersole et al., 2013) | (Belstrom et al., 2014) |
| 3 | (Gursoy et al., 2013) | (Salazar et al., 2013) |

* snow ball means the further search work after full-text screening. We searched the eligible papers from the reference lists of the included papers.

**APPENDICES S5.1** JBI Critical Appraisal Checklist for Randomized Controlled Trials

| **Randomized Controlled Trials** | (Mourao et al., 2013) | (Jones et al., 2013) | (Graziani et al., 2018) |
| --- | --- | --- | --- |
| Randomization for assignment | **+** | **-** | **+** |
| Allocation concealed | **-** | **-** | **+** |
| Groups similar | **+** | **-** | **+** |
| Participants blind | **-** | **-** | **-** |
| Treatment blind | **-** | **-** | **-** |
| Assessors blind | **+** | **+** | **-** |
| Treated identically | **+** | **+** | **+** |
| Follow up complete | **-** | **+** | **+** |
| Analyzed in randomized | **-** | **-** | **-** |
| Measured in the same way | **+** | **+** | **+** |
| Measured in a reliable way | **+** | **-** | **-** |
| Statistical analysis | **+** | **+** | **+** |
| Trial design | **-** | **+** | **+** |
| Percentage of yes | 53.8 % | 46.2 % | 61.5 % |
| **Risk of Bias*** | **substantial** | **substantial** | **moderate** |

* 0%-39% may represent a high risk of bias; 40%-59% may represent a substantial risk of bias; 60%-79% may represent a moderate risk of bias; 80%-100% may represent a low risk of bias. + = Yes, - = No.

**APPENDICES S5.2** JBI Critical Appraisal Checklist for Cohort Studies

| **Cohort Studies** | (Raber-Durlacher et al., 2013) | (Ricardo et al., 2015) | (Lee et al., 2017) |
| --- | --- | --- | --- |
| Similar groups | **-** | **-** | **-** |
| Similar exposures | **+** | **+** | **-** |
| Exposure in a valid way | **-** | **-** | **-** |
| Exposure in a reliable way | **-** | **-** | **-** |
| Confounding identified | **+** | **+** | **+** |
| Strategies for confounding | **+** | **+** | **+** |
| Participants free of the outcome | **-** | **+** | **+** |
| Outcomes measured in a valid way | **+** | **+** | **+** |
| Outcomes measured in a reliable way | **-** | **-** | **-** |
| Follow up time reported and sufficient | **-** | **+** | **+** |
| Follow up complete | **-** | **+** | **+** |
| Strategies to incomplete follow up | **-** | **+** | **-** |
| Appropriate statistical analysis | **+** | **+** | **+** |
| Percentage of yes | 38.4 % | 69.2 % | 53.8 % |
| **Risk of Bias** | **high** | **moderate** | **substantia** |

* 0%-39% may represent a high risk of bias; 40%-59% may represent a substantial risk of bias; 60%-79% may represent a moderate risk of bias;

80%-100% may represent a low risk of bias. + = Yes, - = No.

**APPENDICES S5.3** JBI Critical Appraisal Checklist for Analytical Cross-Sectional Studies

| **Cross-Sectional Studies** | (Gokhale et al., 2013) | (Apatzidou et al., 2013) | (Tabari et al., 2013) | (Zimmermann et al., 2013) | (Kebschull et al., 2013) | (Salazar et al., 2013) | (Gursoy et al., 2013) |
| --- | --- | --- | --- | --- | --- | --- | --- |
| Inclusion criteria defined | **+** | **+** | **+** | **+** | **+** | **+** | **-** |
| Subjects described in detail | **+** | **-** | **-** | **+** | **-** | **+** | **+** |
| Exposure in a valid way | **+** | **+** | **-** | **+** | **-** | **+** | **-** |
| Exposure in a reliable way | **-** | **-** | **-** | **+** | **-** | **+** | **-** |
| Objective condition | **+** | **+** | **+** | **+** | **+** | **+** | **+** |
| Identified confounding factors | **+** | **+** | **+** | **+** | **-** | **-** | **+** |
| Strategies to confounding factors | **+** | **+** | **+** | **+** | **-** | **-** | **+** |
| Outcomes in a valid way | **+** | **+** | **+** | **+** | **+** | **+** | **+** |
| Outcomes in a reliable way | **-** | **-** | **-** | **+** | **-** | **-** | **+** |
| Appropriate statistical analysis | **+** | **+** | **+** | **+** | **+** | **+** | **+** |
| Percentage of yes | 80 % | 70 % | 60 % | 100 % | 40 % | 70 % | 70 % |
| **Risk of Bias** | **low** | **moderate** | **moderate** | **low** | **substantial** | **moderate** | **moderate** |

* 0%-39% may represent a high risk of bias; 40%-59% may represent a substantial risk of bias; 60%-79% may represent a moderate risk of bias; 80%-100% may represent a low risk of bias. + = Yes, - = No.

**APPENDICES S5.3** JBI Critical Appraisal Checklist for Analytical Cross-Sectional Studies (continued)

| **Cross-Sectional Studies** | (Wang et al., 2013) | (Rathnayake et al., 2013) | (Ebersole et al., 2013) | (Duran-Pinedo et al., 2014) | (Beklen and Tsaous Memet, 2014) | (Tabari et al., 2014) | (Schjetlein et al., 2014) |
| --- | --- | --- | --- | --- | --- | --- | --- |
| Inclusion criteria defined | **-** | **+** | **+** | **+** | **+** | **+** | **-** |
| Subjects described in detail | **-** | **+** | **+** | **-** | **-** | **+** | **+** |
| Exposure in a valid way | **-** | **+** | **+** | **-** | **-** | **+** | **+** |
| Exposure in a reliable way | **-** | **+** | **-** | **-** | **-** | **-** | **-** |
| Objective condition | **+** | **-** | **+** | **-** | **+** | **+** | **+** |
| Identified confounding factors | **-** | **+** | **+** | **+** | **+** | **+** | **+** |
| Strategies to confounding factors | **-** | **+** | **+** | **+** | **-** | **+** | **-** |
| Outcomes in a valid way | **+** | **+** | **+** | **+** | **+** | **+** | **+** |
| Outcomes in a reliable way | **-** | **-** | **+** | **-** | **-** | **-** | **+** |
| Appropriate statistical analysis | **-** | **+** | **+** | **+** | **+** | **+** | **+** |
| Percentage of yes | 20 % | 80 % | 90 % | 50 % | 50 % | 80 % | 70 % |
| **Risk of Bias** | **high** | **low** | **low** | **substantial** | **substantial** | **low** | **moderate** |

* 0%-39% may represent a high risk of bias; 40%-59% may represent a substantial risk of bias; 60%-79% may represent a moderate risk of bias; 80%-100% may represent a low risk of bias. + = Yes, - = No.

**APPENDICES S5.3** JBI Critical Appraisal Checklist for Analytical Cross-Sectional Studies (continued)

| **Cross-Sectional Studies** | (Ramirez et al., 2014) | (Mesa et al., 2014) | (Sharma et al., 2014) | (Singh et al., 2014) | (Garneata et al., 2015) | (Torrungruang et al., 2015) | (Lavu et al., 2015) | (Papathanasiou et al., 2014) |
| --- | --- | --- | --- | --- | --- | --- | --- | --- |
| Inclusion criteria defined | **+** | **+** | **+** | **+** | **+** | **-** | **+** | **+** |
| Subjects described in detail | **+** | **+** | **+** | **+** | **-** | **+** | **-** | **+** |
| Exposure in a valid way | **-** | **+** | **-** | **-** | **-** | **-** | **+** | **+** |
| Exposure in a reliable way | **-** | **+** | **-** | **+** | **-** | **-** | **-** | **-** |
| Objective condition | **+** | **+** | **-** | **+** | **+** | **-** | **+** | **+** |
| Identified confounding factors | **+** | **+** | **+** | **+** | **+** | **+** | **+** | **+** |
| Strategies to confounding factors | **+** | **+** | **+** | **+** | **+** | **+** | **+** | **+** |
| Outcomes in a valid way | **+** | **+** | **+** | **+** | **+** | **+** | **+** | **+** |
| Outcomes in a reliable way | **+** | **-** | **-** | **-** | **-** | **-** | **-** | **-** |
| Appropriate statistical analysis | **+** | **+** | **+** | **+** | **+** | **+** | **+** | **+** |
| Percentage of yes | 80 % | 90 % | 60 % | 80 % | 60 % | 50 % | 70 % | 80 % |
| **Risk of Bias** | **low** | **low** | **moderate** | **low** | **moderate** | **substantial** | **moderate** | **low** |

* 0%-39% may represent a high risk of bias; 40%-59% may represent a substantial risk of bias; 60%-79% may represent a moderate risk of bias; 80%-100% may represent a low risk of bias. + = Yes, - = No.

**APPENDICES S5.3** JBI Critical Appraisal Checklist for Analytical Cross-Sectional Studies (continued)

| **Cross-Sectional Studies** | (Ozcan et al., 2015) | (Kirst et al., 2015) | (Kursunlu et al., 2015) | (Chaiyarit et al., 2015) | (Velosa-Porras et al., 2016) | (Prodan et al., 2016) | (Noguera-Julian et al., 2017) | (Saglam et al., 2017) | (Ghallab et al., 2015) |
| --- | --- | --- | --- | --- | --- | --- | --- | --- | --- |
| Inclusion criteria defined | **+** | **+** | **+** | **-** | **+** | **+** | **+** | **+** | **+** |
| Subjects described in detail | **-** | **+** | **-** | **-** | **+** | **+** | **+** | **-** | **+** |
| Exposure in a valid way | **+** | **-** | **-** | **-** | **+** | **-** | **-** | **-** | **+** |
| Exposure in a reliable way | **-** | **-** | **-** | **-** | **-** | **-** | **-** | **+** | **+** |
| Objective condition | **+** | **+** | **+** | **+** | **+** | **+** | **+** | **+** | **+** |
| Identified confounding factors | **+** | **-** | **+** | **-** | **+** | **+** | **+** | **+** | **+** |
| Strategies to confounding factors | **+** | **-** | **+** | **-** | **+** | **+** | **+** | **+** | **+** |
| Outcomes in a valid way | **+** | **+** | **+** | **+** | **+** | **+** | **+** | **+** | **+** |
| Outcomes in a reliable way | **-** | **-** | **-** | **-** | **-** | **-** | **-** | **-** | **-** |
| Appropriate statistical analysis | **+** | **+** | **+** | **+** | **+** | **+** | **+** | **+** | **+** |
| Percentage of yes | 70 % | 50 % | 60 % | 30 % | 80 % | 70 % | 70 % | 70 % | 90 % |
| **Risk of Bias** | **moderate** | **substantial** | **moderate** | **high** | **low** | **moderate** | **moderate** | **moderate** | **low** |

* 0%-39% may represent a high risk of bias; 40%-59% may represent a substantial risk of bias; 60%-79% may represent a moderate risk of bias; 80%-100% may represent a low risk of bias. + = Yes, - = No.

**APPENDICES S5.3** JBI Critical Appraisal Checklist for Analytical Cross-Sectional Studies (continued)

| **Cross-Sectional Studies** | (Wara-aswapati et al., 2013) | (Javed et al., 2014) | (Panezai et al., 2018) | (Huang et al., 2018) | (Kim et al., 2013) | (Pushparani et al., 2014) | (Shetty et al., 2016b) |
| --- | --- | --- | --- | --- | --- | --- | --- |
| Inclusion criteria defined | **-** | **+** | **+** | **-** | **+** | **+** | **+** |
| Subjects described in detail | **+** | **+** | **+** | **+** | **+** | **-** | **-** |
| Exposure in a valid way | **+** | **+** | **+** | **+** | **+** | **+** | **+** |
| Exposure in a reliable way | **-** | **-** | **-** | **-** | **-** | **+** | **-** |
| Objective condition | **+** | **+** | **+** | **+** | **+** | **+** | **+** |
| Identified confounding factors | **+** | **+** | **+** | **+** | **+** | **+** | **+** |
| Strategies to confounding factors | **-** | **+** | **+** | **+** | **+** | **+** | **+** |
| Outcomes in a valid way | **+** | **+** | **+** | **+** | **+** | **+** | **+** |
| Outcomes in a reliable way | **-** | **-** | **-** | **-** | **-** | **-** | **-** |
| Appropriate statistical analysis | **+** | **+** | **+** | **+** | **+** | **+** | **+** |
| Percentage of yes | 60 % | 80 % | 80 % | 70 % | 80 % | 80 % | 70 % |
| **Risk of Bias** | **moderate** | **low** | **low** | **moderate** | **low** | **low** | **moderate** |

* 0%-39% may represent a high risk of bias; 40%-59% may represent a substantial risk of bias; 60%-79% may represent a moderate risk of bias; 80%-100% may represent a low risk of bias. + = Yes, - = No.

**APPENDICES S5.4** JBI Critical Appraisal Checklist for Case Control Studies

| **Case Control Studies** | **(Lourenco et al., 2014)** |
| --- | --- |
| Groups comparable | **-** |
| Cases and controls matched | **+** |
| Same criteria for cases and controls | **+** |
| Exposure in a valid way | **+** |
| Exposure in a reliable way | **+** |
| Exposure in the same way | **+** |
| Confounding factors identified | **-** |
| Strategies for confounding factors | **-** |
| Outcomes in a standard and valid way | **+** |
| Outcomes in a reliable way | **-** |
| Exposure period long enough | **+** |
| Appropriate statistical analysis | **+** |
| Percentage of yes | 66.7 % |
| **Risk of Bias** | **moderate** |

* 0%-39% may represent a high risk of bias; 40%-59% may represent a substantial risk of bias; 60%-79% may represent a moderate risk of bias; 80%-100% may represent a low risk of bias. + = Yes, - = No.

**APPENDICES S5.5** JBI Critical Appraisal Checklist for Quasi-Experimental Studies (non-randomized experimental studies)

| **Quasi-Experimental Studies** | (Sukhtankar et al., 2013) | (Leite et al., 2014) | (Guentsch et al., 2014) | (Hassan et al., 2015) | (Muthu et al., 2015) | (Al-Hamoudi et al., 2018) |
| --- | --- | --- | --- | --- | --- | --- |
| Cause and effect | **+** | **+** | **+** | **+** | **+** | **+** |
| Comparisons similar | **+** | **+** | **-** | **+** | **-** | **+** |
| Similar treatment | **+** | **+** | **+** | **+** | **-** | **+** |
| Control group | **+** | **+** | **+** | **+** | **+** | **+** |
| Multiple measurements of the outcome | **+** | **+** | **+** | **+** | **+** | **+** |
| Follow up complete | **-** | **+** | **-** | **-** | **-** | **+** |
| Follow up described and analyzed | **-** | **-** | **-** | **-** | **+** | **+** |
| Outcomes in the same way | **+** | **+** | **+** | **+** | **+** | **+** |
| Outcomes in a reliable way | **-** | **-** | **+** | **-** | **-** | **-** |
| Appropriate statistical analysis | **+** | **+** | **+** | **+** | **+** | **+** |
| Percentage of yes | 70 % | 80 % | 70 % | 70 % | 60% | 90% |
| **Risk of Bias** | **moderate** | **low** | **moderate** | **moderate** | **moderate** | **low** |

* 0%-39% may represent a high risk of bias; 40%-659% may represent a substantial risk of bias; 60%-79% may represent a moderate risk of bias; 80%-100% may represent a low risk of bias. + = Yes, - = No.

**Appendices S6** Classification tree of included papers


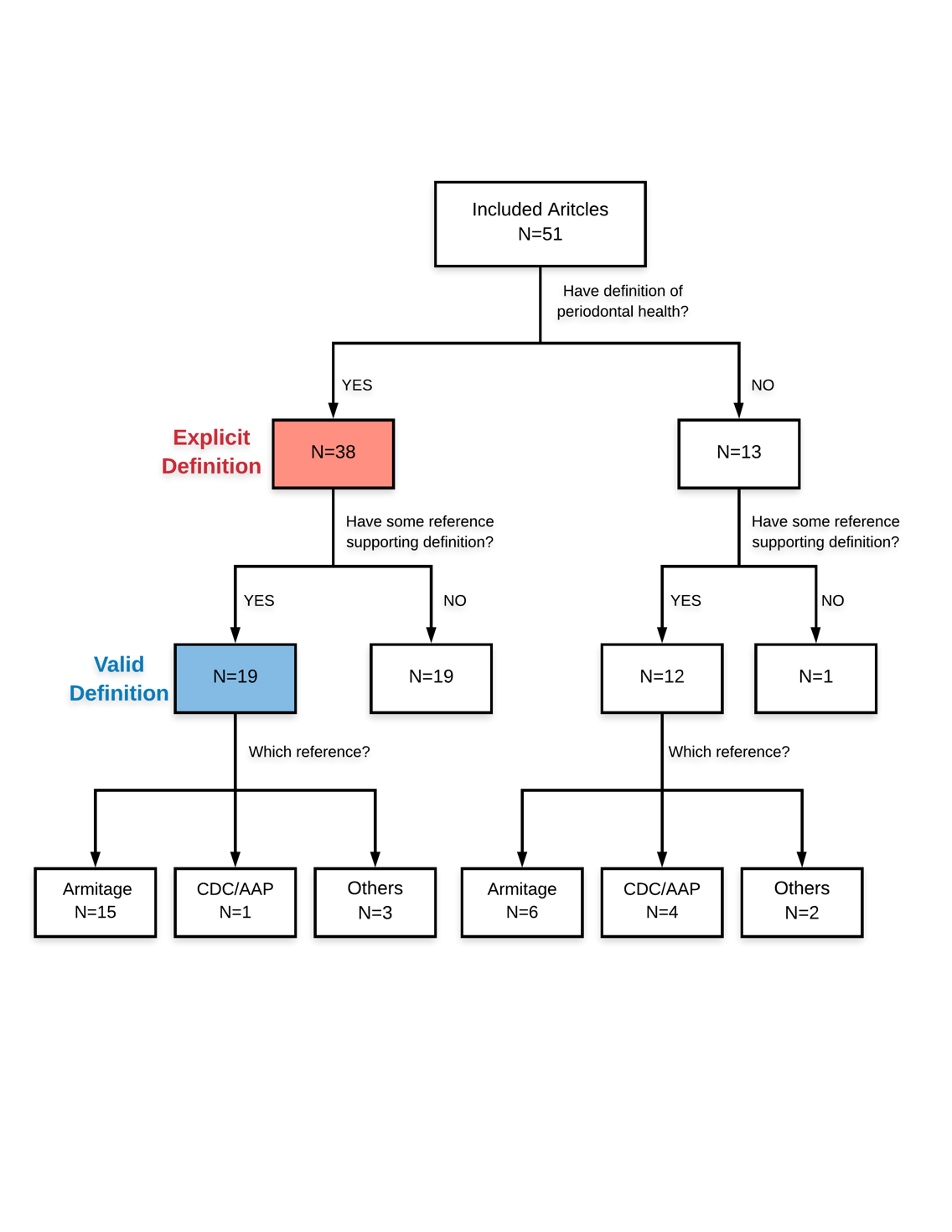


The 38 papers reporting identified definition of periodontal health were regarded as explicit definition. The 19 papers providing identified definition and reference were regarded as valid. Armitage means the classification of periodontal disease proposed by Armitage (Armitage, 1999). CDC/AAP means the case definition of periodontal disease proposed by Page and Eke (Page and Eke, 2007). Others means the other references except from the above two. There are twenty one studies used Armitage to support the definition. Moreover, five studies referred CDC/AAP.

**Appendices S7.1** Definitions of periodontal health according to Armitage (1999) classification.

| **Author, year** | **Clinical Attachment Loss (CAL)** | **Pocket Depth**  **(PD)** | **Bleeding on Probe (BOP)** |
| --- | --- | --- | --- |
| (Tabari et al., 2013) | N.R. | < 3 mm | sites < 10% |
| (Kebschull et al., 2013) | < 4 mm | < 4 mm | NR |
| (Ebersole et al., 2013) | < 2 mm | < 4 mm | sites < 10% |
| (Zimmermann et al., 2013) | < 3 mm | < 3 mm | NR |
| (Gokhale et al., 2013) | < 2 mm | < 4 mm | NR |
| (Tabari et al., 2014) | = 0 | < 3 mm | NR |
| (Lourenco et al., 2014) | < 1 mm | < 3 mm | sites < 10% |
| (Leite et al., 2014) | N.R. | < 4 mm | sites < 10% |
| (Ramirez et al., 2014) | < 3 mm | < 3 mm | sites < 10% |
|  | < 4 mm | < 4 mm | sites < 5% |
| (Chaiyarit et al., 2015) | < 4 mm | < 5 mm | NR |
| (Ghallab et al., 2015) | = 0 mm | < 3 mm | NR |
| (Lavu et al., 2015) | = 0 mm | < 3 mm | NR |
| (Kursunlu et al., 2015) | = 0 mm | < 3 mm | NR |
| (Kirst et al., 2015) | NR | < 3 mm | NR |
| (Huang et al., 2018) | = 0 mm | = 0 | NR |
| (Velosa-Porras et al., 2016) | without disease | without disease | without disease |
| (Lee et al., 2017) | without disease | without disease | without disease |
| (Wara-aswapati et al., 2013) | without disease | without disease | without disease |
| (Pushparani et al., 2014) | without disease | without disease | without disease |
| (Javed et al., 2014) | without disease | without disease | without disease |
| (Al-Hamoudi et al., 2018) | without disease | without disease | without disease |

Chronic Periodontitis defined the periodontal disease as: slight: CAL= 1-2 mm; Moderate: CAL = 3-4 mm; Severe: CAL > 5 mm. (Armitage, 1999) N.R. means not reported.

**Appendices S7.2** Definitions of periodontal health according to CDC/AAP case definition.

| **Author, year** | **Probing Pocket Depth** | **Clinical Attachment Loss** | **Bleeding on Probe** |
| --- | --- | --- | --- |
| (Singh et al., 2014) | PD < 3 mm | CAL = 0 | BOP sites <10% |
| (Ricardo et al., 2015) | without disease | without disease | without disease |
| (Torrungruang et al., 2015) | without disease | without disease | without disease |
| (Hassan et al., 2015) | without disease | without disease | without disease |
| (Noguera-Julian et al., 2017) | without disease | without disease | without disease |

CDC/AAP case definition of periodontal disease: moderate: at least 2 mesial sites with ≥ 4 mm CAL OR at least 2 mesial sites with ≥ 5 mm PD, Severe: at least 2 mesial sites with ≥ 6 mm CAL AND at least 2 mesial sites with ≥ 5 mm PD. (Page and Eke, 2007)

**Appendices S8.1** Rate of explicit periodontal health definition stratified by journal category, study design, risk of bias, and patient resource.

|  | **Publication, N** | **Explicit PdH definition, N (%)** |
| --- | --- | --- |
| **Journal category** |  |  |
| Periodontology journal | 15 | 13(86.7) |
| Dental journal | 15 | 12 (80.0) |
| Medicine journal | 21 | 14 (66.7) |
| **Study design** |  |  |
| RCT | 3 | 3 (100) |
| Non-randomized experimental study | 7 | 6 (85.7) |
| Cohort | 3 | 1 (33.3) |
| Case control | 1 | 1 (100) |
| Cross section | 37 | 28 (75.7) |
| **Risk of bias** |  |  |
| Low | 15 | 11 (73.3) |
| Moderate | 25 | 20 (80.0) |
| Substantial | 8 | 6 (75.0) |
| High | 3 | 2 (66.7) |
| **Patient resource** |  |  |
| Department of periodontology | 11 | 10 (90.9) |
| Dental hospital | 22 | 16 (72.7) |
| General hospital | 9 | 7 (77.8) |
| Population | 9 | 6 (66.7) |

**Appendices S8.2** Rate of valid periodontal health definition stratified by journal category, study design, risk of bias, and patient resource.

|  | **Publication, N** | **Valid PdH definition, N (%)** |
| --- | --- | --- |
| **Journal category** |  |  |
| Periodontology journal | 15 | 6(45.5) |
| Dental journal | 15 | 5 (36.4) |
| Medicine journal | 21 | 7 (31.6) |
| **Study design** |  |  |
| RCT | 3 | 1 (0) |
| Non-randomized experimental study | 7 | 2 (50) |
| Cohort | 3 | 0 (0) |
| Case control | 1 | 1 (100) |
| Cross section | 37 | 14 (38.7) |
| **Risk of bias** |  |  |
| Low | 15 | 8 (63.6) |
| Moderate | 25 | 8 (31.6) |
| Substantial | 8 | 2 (25) |
| High | 3 | 0 (0) |
| **Patient resource** |  |  |
| Department of periodontology | 11 | 5 (45.5) |
| Dental hospital | 22 | 7 (31.8) |
| General hospital | 9 | 4 (44.4) |
| Population | 9 | 2 (22.2) |

**Appendices S9**

References.

Abusleme, L., Dupuy, A. K., Dutzan, N., Silva, N., Burleson, J. A., Strausbaugh, L. D., Gamonal, J. & Diaz, P. I. (2013) The subgingival microbiome in health and periodontitis and its relationship with community biomass and inflammation. *Isme j* **7,** 1016-1025. doi:10.1038/ismej.2012.174.

Al-Hamoudi, N., Abduljabbar, T., Mirza, S., Al-Sowygh, Z. H., Vohra, F., Javed, F. & Akram, Z. (2018) Non-surgical periodontal therapy reduces salivary adipocytokines in chronic periodontitis patients with and without obesity. *J Investig Clin Dent* **9,** e12314. doi:10.1111/jicd.12314.

Apatzidou, A. D., Bakirtzoglou, E., Vouros, I., Karagiannis, V., Papa, A. & Konstantinidis, A. (2013) Association between oral malodour and periodontal disease-related parameters in the general population. *Acta Odontol Scand* **71,** 189-195. doi:10.3109/00016357.2011.654259.

Armitage, G. C. (1999) Development of a classification system for periodontal diseases and conditions. *Ann Periodontol* **4,** 1-6. doi:10.1902/annals.1999.4.1.1.

Beklen, A. & Tsaous Memet, G. (2014) Interleukin-1 superfamily member, interleukin-33, in periodontal diseases. *Biotech Histochem* **89,** 209-214. doi:10.3109/10520295.2013.832800.

Belstrom, D., Fiehn, N. E., Nielsen, C. H., Kirkby, N., Twetman, S., Klepac-Ceraj, V., Paster, B. J. & Holmstrup, P. (2014) Differences in bacterial saliva profile between periodontitis patients and a control cohort. *J Clin Periodontol* **41,** 104-112. doi:10.1111/jcpe.12190.

Biju, T., Shabeer, M. M., Amitha, R., Rajendra, B. P. & Suchetha, K. (2014) Comparative evaluation of serum superoxide dismutase and glutathione levels in periodontally diseased patients: an interventional study. *Indian J Dent Res* **25,** 613-616. doi:10.4103/0970-9290.147105.

Burgess, J. O., Sadid-Zadeh, R., Cakir, D. & Ramp, L. C. (2013) Clinical evaluation of self-etch and total-etch adhesive systems in noncarious cervical lesions: a two-year report. *Operative dentistry* **38,** 477‐487. doi:10.2341/12-355-CR.

Chaiyarit, P., Taweechaisupapong, S., Jaresitthikunchai, J., Phaonakrop, N. & Roytrakul, S. (2015) Comparative evaluation of 5-15-kDa salivary proteins from patients with different oral diseases by MALDI-TOF/TOF mass spectrometry. *Clin Oral Investig* **19,** 729-737. doi:10.1007/s00784-014-1293-3.

Chen, Y. T., Shih, C. J., Ou, S. M., Hung, S. C., Lin, C. H. & Tarng, D. C. (2015) Periodontal Disease and Risks of Kidney Function Decline and Mortality in Older People: A Community-Based Cohort Study. *Am J Kidney Dis* **66,** 223-230. doi:10.1053/j.ajkd.2015.01.010.

Chou, Y. Y., Lai, K. L., Chen, D. Y., Lin, C. H. & Chen, H. H. (2015) Rheumatoid Arthritis Risk Associated with Periodontitis Exposure: A Nationwide, Population-Based Cohort Study. *PLoS One* **10,** e0139693. doi:10.1371/journal.pone.0139693.

Chung, S. D., Tsai, M. C., Huang, C. C., Kao, L. T. & Chen, C. H. (2016) A population-based study on the associations between chronic periodontitis and the risk of cancer. *Int J Clin Oncol* **21,** 219-223. doi:10.1007/s10147-015-0884-6.

Clarkson, J. E., Ramsay, C. R., Averley, P., Bonetti, D., Boyers, D., Campbell, L., Chadwick, G. R., Duncan, A., Elders, A., Gouick, J. & et al. (2013) IQuaD dental trial; improving the quality of dentistry: a multicentre randomised controlled trial comparing oral hygiene advice and periodontal instrumentation for the prevention and management of periodontal disease in dentate adults attending dental primary care. *BMC Oral Health* **13,** 58. doi:10.1186/1472-6831-13-58.

Duran-Pinedo, A. E., Chen, T., Teles, R., Starr, J. R., Wang, X., Krishnan, K. & Frias-Lopez, J. (2014) Community-wide transcriptome of the oral microbiome in subjects with and without periodontitis. *Isme j* **8,** 1659-1672. doi:10.1038/ismej.2014.23.

Ebersole, J. L., Schuster, J. L., Stevens, J., Dawson, D., 3rd, Kryscio, R. J., Lin, Y., Thomas, M. V. & Miller, C. S. (2013) Patterns of salivary analytes provide diagnostic capacity for distinguishing chronic adult periodontitis from health. *J Clin Immunol* **33,** 271-279. doi:10.1007/s10875-012-9771-3.

Fernandes, L. O., Mota, C., de Melo, L. S. A., da Costa Soares, M. U. S., da Silva Feitosa, D. & Gomes, A. S. L. (2017) In vivo assessment of periodontal structures and measurement of gingival sulcus with Optical Coherence Tomography: a pilot study. *J Biophotonics* **10,** 862-869. doi:10.1002/jbio.201600082.

Garneata, L., Slusanschi, O., Preoteasa, E., Corbu-Stancu, A. & Mircescu, G. (2015) Periodontal status, inflammation, and malnutrition in hemodialysis patients - is there a link? *J Ren Nutr* **25,** 67-74. doi:10.1053/j.jrn.2014.07.004.

Ghallab, N. A., Amr, E. M. & Shaker, O. G. (2015) Expression of Leptin and Visfatin in Gingival Tissues of Chronic Periodontitis With and Without Type 2 Diabetes Mellitus: A Study Using Enzyme-Linked Immunosorbent Assay and Real-Time Polymerase Chain Reaction. *J Periodontol* **86,** 882-889. doi:10.1902/jop.2015.140434.

Gokhale, N. H., Acharya, A. B., Patil, V. S., Trivedi, D. J. & Thakur, S. L. (2013) A short-term evaluation of the relationship between plasma ascorbic acid levels and periodontal disease in systemically healthy and type 2 diabetes mellitus subjects. *J Diet Suppl* **10,** 93-104. doi:10.3109/19390211.2013.790332.

Gong, Y., Lu, J., Ding, X. & Yu, Y. (2014) Effect of adjunctive roxithromycin therapy on interleukin-1β, transforming growth factor-β1 and vascular endothelial growth factor in gingival crevicular fluid of cyclosporine A-treated patients with gingival overgrowth. *Journal of periodontal research* **49,** 448‐457. doi:10.1111/jre.12123.

Graziani, F., Palazzolo, A., Gennai, S., Karapetsa, D., Giuca, M. R., Cei, S., Filice, N., Petrini, M. & Nisi, M. (2018) Interdental plaque reduction after use of different devices in young subjects with intact papilla: A randomized clinical trial. *Int J Dent Hyg* **16,** 389-396. doi:10.1111/idh.12318.

Guentsch, A., Pfister, W., Cachovan, G., Raschke, G., Kuepper, H., Schaefer, O. & Eick, S. (2014) Oral prophylaxis and its effects on halitosis-associated and inflammatory parameters in patients with chronic periodontitis. *Int J Dent Hyg* **12,** 199-207. doi:10.1111/idh.12063.

Gursoy, U. K., Kononen, E., Huumonen, S., Tervahartiala, T., Pussinen, P. J., Suominen, A. L. & Sorsa, T. (2013) Salivary type I collagen degradation end-products and related matrix metalloproteinases in periodontitis. *J Clin Periodontol* **40,** 18-25. doi:10.1111/jcpe.12020.

Hassan, S. H., El-Refai, M. I., Ghallab, N. A., Kasem, R. F. & Shaker, O. G. (2015) Effect of periodontal surgery on osteoprotegerin levels in gingival crevicular fluid, saliva, and gingival tissues of chronic periodontitis patients. *Dis Markers* **2015,** 341259. doi:10.1155/2015/341259.

Herman, M., Golasik, M., Piekoszewski, W., Walas, S., Napierala, M., Wyganowska-Swiatkowska, M., Kurhanska-Flisykowska, A., Wozniak, A. & Florek, E. (2016) Essential and Toxic Metals in Oral Fluid-a Potential Role in the Diagnosis of Periodontal Diseases. *Biol Trace Elem Res* **173,** 275-282. doi:10.1007/s12011-016-0660-0.

Hsu, C. C., Hsu, Y. C., Chen, H. J., Lin, C. C., Chang, K. H., Lee, C. Y., Chong, L. W. & Kao, C. H. (2015) Association of Periodontitis and Subsequent Depression: A Nationwide Population-Based Study. *Medicine (Baltimore)* **94,** e2347. doi:10.1097/md.0000000000002347.

Huang, B., Dai, Q. & Huang, S. G. (2018) Expression of Tolllike receptor 4 on mast cells in gingival tissues of human chronic periodontitis. *Mol Med Rep* **17,** 6731-6735. doi:10.3892/mmr.2018.8648.

Huang, Y. F., Chang, C. T., Liu, S. P., Muo, C. H., Tsai, C. H., Hong, H. H., Shen, Y. F. & Wu, C. Z. (2016) The Impact of Oral Hygiene Maintenance on the Association Between Periodontitis and Osteoporosis: A Nationwide Population-Based Cross Sectional Study. *Medicine (Baltimore)* **95,** e2348. doi:10.1097/md.0000000000002348.

Javed, F., Ahmed, H. B., Saeed, A., Mehmood, A. & Bain, C. (2014) Whole salivary interleukin-6 and matrix metalloproteinase-8 levels in patients with chronic periodontitis with and without prediabetes. *J Periodontol* **85,** e130-135. doi:10.1902/jop.2013.130514.

Jones, C., Macfarlane, T. V., Milsom, K. M., Ratcliffe, P., Wyllie, A. & Tickle, M. (2013) Patient perceptions regarding benefits of single visit scale and polish: a randomised controlled trial. *BMC Oral Health* **13,** 50. doi:10.1186/1472-6831-13-50.

Kebschull, M., Guarnieri, P., Demmer, R. T., Boulesteix, A. L., Pavlidis, P. & Papapanou, P. N. (2013) Molecular differences between chronic and aggressive periodontitis. *J Dent Res* **92,** 1081-1088. doi:10.1177/0022034513506011.

Kim, E. K., Lee, S. G., Choi, Y. H., Won, K. C., Moon, J. S., Merchant, A. T. & Lee, H. K. (2013) Association between diabetes-related factors and clinical periodontal parameters in type-2 diabetes mellitus. *BMC oral health* **13,** 64. doi:10.1186/1472-6831-13-64.

Kirst, M. E., Li, E. C., Alfant, B., Chi, Y. Y., Walker, C., Magnusson, I. & Wang, G. P. (2015) Dysbiosis and alterations in predicted functions of the subgingival microbiome in chronic periodontitis. *Appl Environ Microbiol* **81,** 783-793. doi:10.1128/aem.02712-14.

Kongstad, J., Ekstrand, K., Qvist, V., Christensen, L. B., Cortsen, B., Gronbaek, M., Holm-Pedersen, P., Holmstrup, P., Bardow, A., Twetman, S. & Fiehn, N. E. (2013) Findings from the oral health study of the Danish Health Examination Survey 2007-2008. *Acta Odontol Scand* **71,** 1560-1569. doi:10.3109/00016357.2013.776701.

Konig, M. F., Abusleme, L., Reinholdt, J., Palmer, R. J., Teles, R. P., Sampson, K., Rosen, A., Nigrovic, P. A., Sokolove, J., Giles, J. T., Moutsopoulos, N. M. & Andrade, F. (2016) Aggregatibacter actinomycetemcomitans-induced hypercitrullination links periodontal infection to autoimmunity in rheumatoid arthritis. *Sci Transl Med* **8,** 369ra176. doi:10.1126/scitranslmed.aaj1921.

Kursunlu, S. F., Ozturk, V. O., Han, B., Atmaca, H. & Emingil, G. (2015) Gingival crevicular fluid interleukin-36beta (-1F8), interleukin-36gamma (-1F9) and interleukin-33 (-1F11) levels in different periodontal disease. *Arch Oral Biol* **60,** 77-83. doi:10.1016/j.archoralbio.2014.08.021.

Laine, M. L., Moustakis, V., Koumakis, L., Potamias, G. & Loos, B. G. (2013) Modeling susceptibility to periodontitis. *J Dent Res* **92,** 45-50. doi:10.1177/0022034512465435.

Lavu, V., Venkatesan, V., Venkata Kameswara Subrahmanya Lakkakula, B., Venugopal, P., Paul, S. F. & Rao, S. R. (2015) Polymorphic regions in the interleukin-1 gene and susceptibility to chronic periodontitis: a genetic association study. *Genet Test Mol Biomarkers* **19,** 175-181. doi:10.1089/gtmb.2014.0275.

Lee, J. H., Oh, J. Y., Youk, T. M., Jeong, S. N., Kim, Y. T. & Choi, S. H. (2017) Association between periodontal disease and non-communicable diseases: A 12-year longitudinal health-examinee cohort study in South Korea. *Medicine (Baltimore)* **96,** e7398. doi:10.1097/md.0000000000007398.

Leite, A. C., Carneiro, V. M. & Guimaraes Mdo, C. (2014) Effects of periodontal therapy on C-reactive protein and HDL in serum of subjects with periodontitis. *Rev Bras Cir Cardiovasc* **29,** 69-77.

Loozen, G., Ozcelik, O., Boon, N., De Mol, A., Schoen, C., Quirynen, M. & Teughels, W. (2014) Inter-bacterial correlations in subgingival biofilms: a large-scale survey. *J Clin Periodontol* **41,** 1-10. doi:10.1111/jcpe.12167.

Lourenco, T. G., Heller, D., Silva-Boghossian, C. M., Cotton, S. L., Paster, B. J. & Colombo, A. P. (2014) Microbial signature profiles of periodontally healthy and diseased patients. *J Clin Periodontol* **41,** 1027-1036. doi:10.1111/jcpe.12302.

Mdala, I., Olsen, I., Haffajee, A. D., Socransky, S. S., Thoresen, M. & de Blasio, B. F. (2014) Comparing clinical attachment level and pocket depth for predicting periodontal disease progression in healthy sites of patients with chronic periodontitis using multi-state Markov models. *J Clin Periodontol* **41,** 837-845. doi:10.1111/jcpe.12278.

Mesa, F., Magan-Fernandez, A., Munoz, R., Papay-Ramirez, L., Poyatos, R., Sanchez-Fernandez, E., Galindo-Moreno, P. & Rodriguez-Barranco, M. (2014) Catecholamine metabolites in urine, as chronic stress biomarkers, are associated with higher risk of chronic periodontitis in adults. *J Periodontol* **85,** 1755-1762. doi:10.1902/jop.2014.140209.

Mourao, L. C., Moutinho, H. & Canabarro, A. (2013) Additional benefits of homeopathy in the treatment of chronic periodontitis: a randomized clinical trial. *Complement Ther Clin Pract* **19,** 246-250. doi:10.1016/j.ctcp.2013.05.002.

Muthu, J., Muthanandam, S., Mahendra, J., Namasivayam, A., John, L. & Logaranjini, A. (2015) Effect of Nonsurgical Periodontal Therapy on the Glycaemic Control of Nondiabetic Periodontitis Patients: A Clinical Biochemical Study. *Oral Health Prev Dent* **13,** 261-266. doi:10.3290/j.ohpd.a32995.

Noguera-Julian, M., Guillen, Y., Peterson, J., Reznik, D., Harris, E. V., Joseph, S. J., Rivera, J., Kannanganat, S., Amara, R., Nguyen, M. L., Mutembo, S., Paredes, R., Read, T. D. & Marconi, V. C. (2017) Oral microbiome in HIV-associated periodontitis. *Medicine (Baltimore)* **96,** e5821. doi:10.1097/MD.0000000000005821.

Novakovic, N., Todorovic, T., Rakic, M., Milinkovic, I., Dozic, I., Jankovic, S., Aleksic, Z. & Cakic, S. (2014) Salivary antioxidants as periodontal biomarkers in evaluation of tissue status and treatment outcome. *Journal of periodontal research* **49,** 129‐136. doi:10.1111/jre.12088.

Ozcan, E., Saygun, N. I., Serdar, M. A. & Kurt, N. (2015) Evaluation of the salivary levels of visfatin, chemerin, and progranulin in periodontal inflammation. *Clin Oral Investig* **19,** 921-928. doi:10.1007/s00784-014-1308-0.

Page, R. C. & Eke, P. I. (2007) Case definitions for use in population-based surveillance of periodontitis. *J Periodontol* **78,** 1387-1399. doi:10.1902/jop.2007.060264.

Paknejad, M., Sattari, M., Roozbahani, Z., Ershadi, M. & Mehrfard, A. (2016) Relationships between High-mobility Group Protein B1 and Triggering Receptor Expressed on Myeloid Cells Concentrations in Gingival Crevicular Fluid and Chronic Periodontitis. *Iran J Allergy Asthma Immunol* **15,** 381-385.

Panezai, J., Ghaffar, A., Altamash, M., Engstrom, P. E. & Larsson, A. (2018) Periodontal disease influences osteoclastogenic bone markers in subjects with and without rheumatoid arthritis. *Plos one* **13,** e0197235. doi:10.1371/journal.pone.0197235.

Papathanasiou, E., Teles, F., Griffin, T., Arguello, E., Finkelman, M., Hanley, J. & Theoharides, T. C. (2014) Gingival crevicular fluid levels of interferon-gamma, but not interleukin-4 or -33 or thymic stromal lymphopoietin, are increased in inflamed sites in patients with periodontal disease. *J Periodontal Res* **49,** 55-61. doi:10.1111/jre.12078.

Pesevska, S., Gjorgoski, I., Ivanovski, K., Soldatos, N. K. & Angelov, N. (2017) The effect of low-level diode laser on COX-2 gene expression in chronic periodontitis patients. *Lasers in medical science* **32,** 1463‐1468. doi:10.1007/s10103-017-2231-9.

Prodan, A., Brand, H., Imangaliyev, S., Tsivtsivadze, E., van der Weijden, F., de Jong, A., Paauw, A., Crielaard, W., Keijser, B. & Veerman, E. (2016) A Study of the Variation in the Salivary Peptide Profiles of Young Healthy Adults Acquired Using MALDI-TOF MS. *PLoS One* **11,** e0156707. doi:10.1371/journal.pone.0156707.

Pushparani, D. S., Anandan, S. N. & Theagarayan, P. (2014) Serum zinc and magnesium concentrations in type 2 diabetes mellitus with periodontitis. *J Indian Soc Periodontol* **18,** 187-193. doi:10.4103/0972-124x.131322.

Raber-Durlacher, J. E., Laheij, A. M., Epstein, J. B., Epstein, M., Geerligs, G. M., Wolffe, G. N., Blijlevens, N. M. & Donnelly, J. P. (2013) Periodontal status and bacteremia with oral viridans streptococci and coagulase negative staphylococci in allogeneic hematopoietic stem cell transplantation recipients: a prospective observational study. *Support Care Cancer* **21,** 1621-1627. doi:10.1007/s00520-012-1706-2.

Ramirez, J. H., Parra, B., Gutierrez, S., Arce, R. M., Jaramillo, A., Ariza, Y. & Contreras, A. (2014) Biomarkers of cardiovascular disease are increased in untreated chronic periodontitis: a case control study. *Aust Dent J* **59,** 29-36. doi:10.1111/adj.12139.

Rathnayake, N., Akerman, S., Klinge, B., Lundegren, N., Jansson, H., Tryselius, Y., Sorsa, T. & Gustafsson, A. (2013) Salivary biomarkers of oral health: a cross-sectional study. *J Clin Periodontol* **40,** 140-147. doi:10.1111/jcpe.12038.

Ricardo, A. C., Athavale, A., Chen, J., Hampole, H., Garside, D., Marucha, P. & Lash, J. P. (2015) Periodontal disease, chronic kidney disease and mortality: results from the third National Health and Nutrition Examination Survey. *BMC Nephrol* **16,** 97. doi:10.1186/s12882-015-0101-x.

Saglam, M., Koseoglu, S., Aral, C. A., Savran, L., Pekbagriyanik, T. & Cetinkaya, A. (2017) Increased levels of interleukin-33 in gingival crevicular fluids of patients with chronic periodontitis. *Odontology* **105,** 184-190. doi:10.1007/s10266-016-0259-0.

Salazar, M. G., Jehmlich, N., Murr, A., Dhople, V. M., Holtfreter, B., Hammer, E., Volker, U. & Kocher, T. (2013) Identification of periodontitis associated changes in the proteome of whole human saliva by mass spectrometric analysis. *J Clin Periodontol* **40,** 825-832. doi:10.1111/jcpe.12130.

Saliasi, I., Llodra, J. C., Bravo, M., Tramini, P., Dussart, C., Viennot, S. & Carrouel, F. (2018) Effect of a Toothpaste/Mouthwash Containing Carica papaya Leaf Extract on Interdental Gingival Bleeding: A Randomized Controlled Trial. *Int J Environ Res Public Health* **15**. doi:10.3390/ijerph15122660.

Schjetlein, A. L., Jorgensen, M. E., Lauritzen, T. & Pedersen, M. L. (2014) Periodontal status among patients with diabetes in Nuuk, Greenland. *Int J Circumpolar Health* **73,** 26093. doi:10.3402/ijch.v73.26093.

Sharma, A., Astekar, M., Metgud, R., Soni, A., Verma, M. & Patel, S. (2014) A study of C-reactive protein, lipid metabolism and peripheral blood to identify a link between periodontitis and cardiovascular disease. *Biotech Histochem* **89,** 577-582. doi:10.3109/10520295.2014.918280.

Shetty, A., Bhandary, R., Thomas, B. & Ramesh, A. (2016) A Comparative Evaluation of Serum Magnesium in Diabetes Mellitus Type 2 Patients with and without Periodontitis - A Clinico-biochemical Study. *J Clin Diagn Res* **10,** Zc59-zc61. doi:10.7860/jcdr/2016/21063.9078.

Singh, N., Chander Narula, S., Kumar Sharma, R., Tewari, S. & Kumar Sehgal, P. (2014) Vitamin E supplementation, superoxide dismutase status, and outcome of scaling and root planing in patients with chronic periodontitis: a randomized clinical trial. *J Periodontol* **85,** 242-249. doi:10.1902/jop.2013.120727.

Sukhtankar, L., Kulloli, A., Kathariya, R. & Shetty, S. (2013) Effect of non-surgical periodontal therapy on superoxide dismutase levels in gingival tissues of chronic periodontitis patients: a clinical and spectophotometric analysis. *Dis Markers* **34,** 305-311. doi:10.3233/dma-130978.

Tabari, Z. A., Azadmehr, A., Nohekhan, A., Naddafpour, N. & Ghaedi, F. B. (2014) Salivary visfatin concentrations in patients with chronic periodontitis. *J Periodontol* **85,** 1081-1085. doi:10.1902/jop.2013.130388.

Tabari, Z. A., Azadmehr, A., Tabrizi, M. A., Hamissi, J. & Ghaedi, F. B. (2013) Salivary soluble receptor activator of nuclear factor kappa B ligand/osteoprotegerin ratio in periodontal disease and health. *J Periodontal Implant Sci* **43,** 227-232. doi:10.5051/jpis.2013.43.5.227.

Toregeani, J. F., Nassar, C. A., Nassar, P. O., Toregeani, K. M., Gonzatto, G. K., Vendrame, R., Castilhos, J. S., Rotta, L. S., Reinheimer, A. C., Longoni, A. & Barcella, M. W. (2016) Evaluation of periodontitis treatment effects on carotid intima-media thickness and expression of laboratory markers related to atherosclerosis. *Gen Dent* **64,** 55-62.

Torrungruang, K., Jitpakdeebordin, S., Charatkulangkun, O. & Gleebbua, Y. (2015) Porphyromonas gingivalis, Aggregatibacter actinomycetemcomitans, and Treponema denticola / Prevotella intermedia Co-Infection Are Associated with Severe Periodontitis in a Thai Population. *PLoS One* **10,** e0136646. doi:10.1371/journal.pone.0136646.

Valor, L. O., Norton, I. K. R., Koldsland, O. C., Aass, A. M., Grjibovski, A. M. & Preus, H. R. (2018) The plaque and gingivitis inhibiting capacity of a commercially available mouthwash containing essential oils and ethyl lauroyl arginate. A randomized clinical trial. *Acta Odontol Scand* **76,** 241-246. doi:10.1080/00016357.2017.1412499.

Vavricka, S. R., Manser, C. N., Hediger, S., Vogelin, M., Scharl, M., Biedermann, L., Rogler, S., Seibold, F., Sanderink, R., Attin, T., Schoepfer, A., Fried, M., Rogler, G. & Frei, P. (2013) Periodontitis and gingivitis in inflammatory bowel disease: a case-control study. *Inflamm Bowel Dis* **19,** 2768-2777. doi:10.1097/01.MIB.0000438356.84263.3b.

Velosa-Porras, J., Escobar-Arregoces, F., Latorre-Uriza, C., Ferro-Camargo, M. B., Ruiz, A. J. & Uriza-Carrasco, L. F. (2016) Association between periodontal disease and endothelial dysfunction in smoking patients. *Acta Odontol Latinoam* **29,** 29-35.

Wang, J., Qi, J., Zhao, H., He, S., Zhang, Y., Wei, S. & Zhao, F. (2013) Metagenomic sequencing reveals microbiota and its functional potential associated with periodontal disease. *Sci Rep* **3,** 1843. doi:10.1038/srep01843.

Wara-aswapati, N., Chayasadom, A., Surarit, R., Pitiphat, W., Boch, J. A., Nagasawa, T., Ishikawa, I. & Izumi, Y. (2013) Induction of toll-like receptor expression by Porphyromonas gingivalis. *J Periodontol* **84,** 1010-1018. doi:10.1902/jop.2012.120362.

Zimmermann, G. S., Bastos, M. F., Dias Goncalves, T. E., Chambrone, L. & Duarte, P. M. (2013) Local and circulating levels of adipocytokines in obese and normal weight individuals with chronic periodontitis. *J Periodontol* **84,** 624-633. doi:10.1902/jop.2012.120254.
